# Quality assessment of medicinal material Daqingye and Banlangen from *Isatis tinctoria* Fort. reveals widespread substitution with *Strobilanthes* species

**DOI:** 10.1371/journal.pone.0323084

**Published:** 2025-05-07

**Authors:** Hiu-Lam Ngai, Sau-Wan Cheng, Tin-Sum Tse, Hung Kay Lee, Pang-Chui Shaw

**Affiliations:** 1 School of Life Sciences, The Chinese University of Hong Kong, Shatin, N.T., Hong Kong, China; 2 LDS YYC R&D Centre for Chinese Medicine and State Key Laboratory of Research on Bioactivities and Clinical Applications of Medicinal Plants, The Chinese University of Hong Kong, Shatin, N.T., Hong Kong, China; 3 Department of Chemistry, The Chinese University of Hong Kong, Shatin, N.T., Hong Kong, China.; Università degli Studi di Milano, ITALY

## Abstract

**Background:**

Isatidis Folium (Daqingye, DQY) and Isatidis Radix (Banlangen, BLG) are the leaf and root of the plant *Isatis tinctoria* Fort. (syn. *Isatis indigotica* Fort.), commonly prescribed for detoxification, and the inhibition of viral and oxidative activities. Given their widespread use, we set forth to investigate the authenticity and chemical composition of DQY and BLG samples obtained from eighteen administrative districts in the Hong Kong market.

**Methods:**

The present study screened the identities and chemical composition of DQY and BLG through molecular authentication and HPLC methods, respectively. Molecular authentication utilized DNA barcoding, focusing on nuclear ribosomal and chloroplast regions. The HPLC methods were conducted in accordance with the Hong Kong Chinese Materia Medica Standards (HKCMMS).

**Results:**

We found that only one sample was genuine according to the species definition in the Chinese Pharmacopoeia and HKCMMS for both herbs. The chemical composition of the adulterated and the genuine samples were completely different, that the adulterant samples did not have the standard chemical markers epigoitrin and indirubin of Banlangen and Daqingye as listed in the Chinese Pharmacopoeia.

**Conclusion:**

Our investigation underscores the widespread substitution by *Strobilanthes* (Nanbanlangen and Nandaqingye) species, mainly due to the preference of use of this herb in southern China. The adulteration of Daqingye by *Blumea balsamifera* (Ainaxiang) was probably due to mislabeling in the herb shop, though the error might have originated from the supplying sources. We recommend providing education on the necessity of using authentic Daqingye and Banlangen, especially in combined regimens, to standardize treatment effects. More education is also needed on the morphological differentiation of Banlangen from Nanbanlangen and Daqingye from Nandaqingye. The implementation of a track-and-trace system is strongly recommended to prevent and deter incorrect supply chain practices that lead to substitution or adulteration.

## Introduction

Isatidis Folium (Daqingye, DQY) and Isatidis Radix (Banlangen, BLG), the leaf and root of the plant *Isatis tinctoria* Fort. (also known as *Isatis indigotica* Fort. or Indigo Woad), belong to the family Brassicaceae. They have been utilized as medicines and are listed in the Chinese Pharmacopoeia for their heat-clearing and detoxification properties in Southeast Asia, China, and India for a long history [1]. Humid environment near the edges of mountain forests is facilitative to the growth of the medicinal plant [2]. Besides medicinal use, the plant serves as a traditional source for extracting indigo, a blue dye, in Europe, North America and Asia [3]. Warm weather and dryer conditions without too much water in soil can facilitate the production. Thus, it is suitable for growing the plant in loose, fertile sandy loam soil, without strict requirements for climate and soil conditions in Central Europe [4] and Northern and Central part of China.

### Botany and production

BLG has been utilized since the Song Dynasty (around 1000 AD). The morphology of BLG is cylindrical, twisted lightly, 10–20 cm long and 0.5–1 cm in diameter. The color is lightly grayish or brownish yellow, with wrinkles, protrusions and root marks. The head of root is a bit enlarged, with remains of leaf stalks arranged circularly, in dark-green or blackish-brown color. It is slightly solid and soft in texture, and sweet and bitter in taste [5]. DQY was originally classified as blue-colored plants from several species in ancient dynasties. Today, the Chinese Pharmacopoeia defines DQY solely as *Isatis tinctoria* Fort. (or *Isatis indigotica* Fort.), both referring to the same species [6]. *Polygonum tinctorium* Ait. and *Clerodendrum cyrtophyllum* Turcz., which were previously recognized as other species of DQY, are now excluded. For consistency, we will refer to the species as *I. tinctoria* throughout this manuscript. When flattened, the intact leaves of DQY are oblong or oblanceolate in shape, measured 0.05–0.2 m long and 15–70 mm wide. The upper surface is dark grayish-green, with some slightly raised darker spots. The apex is obtuse, the margin is entire or slightly wavy, and the base is narrowly elongated into a wing-like petiole. The petiole is 4–10 cm long and light brownish-yellow. The DQY has a loose, crisp texture, a faint aroma, and a sour, bitter, and astringent taste [1].

The main production locations of the *I. tinctoria* are Gansu, Northeast China, Henan, and Anhui in Northern and Central China, where Gansu is the largest production area, with an average annual production of around 7,000 tons [7]. DQY, *I. tinctoria*, is generally harvested 2–3 times in summer and autumn. After harvesting the DQY, the roots of the medicinal plant - *I. tinctoria* become thinner and the stem bases elongate. *I. tinctoria* roots (BLG) harvested more than twice are longer, with more pronounced leaf base remnants and wart-like protrusions on the upper part of the roots [8]. The contradictory supply and demand of plant growth nutrients have led to the yields of BLG and DQY mutually antagonistic. The harvest time of DQY has a great impact on the yield and quality of BLG. Planning and management of the harvesting is crucial to the chemical quality of DQY and BLG, besides variations across closely-related species.

### Ethnopharmacology

BLG was a drug with a long history of use in China [9–11]. In the past fifty years, more than 20 provinces in China, including Anhui, Hebei, Henan, Jiangsu, and Shaanxi [2], have started expanding the production of BLG. BLG products have then been manufactured on a large scale as granules, tablets, and injections, gradually becoming a widely used pharmaceutical drug [12]. Today, BLG has become a frequently applied medicinal herb. BLG has found widespread applications, including dental and oral health, beauty products, and disinfection products. Examples include herbal mouthwashes, floral teas, and hand sanitizers - all of which contain BLG for cleansing and disinfection [13,14].

DQY is a popular herbal medicine in many Asian countries. This popularity is due to its immunomodulatory and antipyretic effects, as well as its antiviral efficacy in treating influenza, measles, acute infectious hepatitis, dysentery, acute gastroenteritis, and acute pneumonia [15]. DQY is also considered a promising drug for the treatment of skin diseases due to its rich anti-inflammatory and antioxidant components. DQY is also a component of beauty products, as it was proved to possess excellent anti-wrinkle activity [16,17]. BLG and/or DQY can also help other herbs better exerting their effects. Classical prescriptions showed long history of such combinations [18].

### Chemical content and clinical uses

BLG and DQY are bitter, cool, and acting on the heart and stomach channels. Since the two herbs can treat lung heat, they are often prescribed for treating flu-related symptoms such as fever and sore throat [18]. The proprietary Chinese medicines Lianhuaqingwen Capsule [12] and Taiwan Chingguan Yihau [19], which are used for treating COVID-19, contain BLG. Experimental data showed BLG and DQY could inhibit virus [20], relieve inflammation [21] and regulate immune functions [22], by their rich chemical compounds.

Due to being the same medicinal plant, chemicals of BLG and DQY are similar and they thus give similar pharmacological activities. However, there are differences in the alkaloid and lignan contents of BLG and DQY, such as indican, indirubin, epigoitrin, 4(3H)-quinazolinone, clemastanin B, and isatindigotindolines A-D [18]. This leads to the different treatment effects of BLG and DQY. BLG is more potent for inhibiting virus and regulating immune factors, while DQY is more effective to inhibit oxidant activities [18]. DQY is effective at blood clearing and spot removing, making it suitable for treating skin rashes and inflammation caused by heat toxins, while BLG is primarily detoxifying and mainly applied for cold, flu, and sore throat. BLG has thus been more frequently included in the treatment regimen of epidemic viral infections such as COVID-19 [12].

### Objectives of this study

Given the significance of BLG and DQY in both single use and combined regimen, together with the unknowing quality and authenticity of these two herbs circulating in the market, this study set forth to reveal the biomarker content and the species information of the samples of these two herbs collected from the eighteen administrative districts in Hong Kong.

## Materials and methods

### Samples studied

One hundred and thirteen grams of each sample was collected from each of the eighteen districts in Hong Kong, coded and stored in Li Dak Sum Yip Yio Chin R&D Centre for Chinese Medicine, The Chinese University of Hong Kong (Table 1). Samples obtained from Wan Chai were of two different species claimed by the retailers, and coded as T5542 and T5543 by us. No permit was required to obtain the samples for the investigation, as they are common medicinal material freely available in the market.

**Table 1:** Samples of Banlangen and Daqingye.

#	Administrative district	Daqingye Code	Banlangen Code
1	Wong Tai Sin District	T5529	T5572
2	Sham Shui Po District	T5530	T5560
3	Yau Tsim Mong District	T5532	T5624
4	Kwun Tong District	T5533	T5564
5	Kowloon City District	T5536	T5557
6	Tai Po District	T5537	T5565
7	Shatin District	T5538	T5569
8	North District	T5539	T5567
9	Sai Kung District	T5540	T5562
10	Eastern District	T5541	T5566
11	Wan Chai District	T5542, T5543	T5561
12	Southern District	T5544	T5573
13	Central and Western District	T5535	T5558
14	Islands District	T5547	T5559
15	Kwai Tsing District	T5531	T5563
16	Tsuen Wan District	T5549	T5568
17	Yuen Long District	T5550	T5571
18	Tuen Mun District	T5551	T5570

We initially examined the samples according to the morphological characteristics outlined in the Chinese Pharmacopoeia and found that most samples did not match the description, suggesting many samples were not BLG or DQY. Images that depict the organoleptic characteristics and morphological identities of samples are shown in the S1 File. We subsequently assessed their authenticity and analyzed the chemical compounds using our standard operating procedures for DNA barcoding and HPLC in the laboratory.

### DNA authentication

#### DNA extraction.

Two hundred milligrams of each sample were physically sheared into fine powder and extracted using a broad-spectrum plant rapid genomic DNA extraction kit (Biomed, China). Fifty microliters of pre-warmed autoclaved Milli-Q ultrapure water were used to recover the purified DNA.

#### PCR and sequencing.

PCR was conducted with universal primers at the *rbcL*, *matK*, *psbA-trnH*, or *ITS2* regions to identify the species of BLG and DQY samples obtained from herb shops. The primers and PCR protocols are listed in S2 File. After PCR amplification, agarose gel electrophoresis was performed and the amplicons were gel purified, and Sanger sequenced by BGI, Hong Kong, China. A reverse complement of the reverse sequence was produced and combined with the forward sequence to produce the consensus sequence of each sample using the BioEdit Sequence Alignment Editor. The consensus sequences were aligned by ClustalW multiple alignment to see differences in nucleotides. The sequences were then queried in GenBank database via BLASTN, NCBI to obtain the identities at highest similarity and coverage, and longest matching accession length. PCR was conducted three times to ensure correct species identification at different DNA regions.

### HPLC analysis

#### Epigoitrin extraction from Banlangen and HPLC assay.

BLG samples were extracted according to the Hong Kong Chinese Materia Medica Standards (HKCMMS, https://www.cmro.gov.hk/files/hkcmms/vol4/pdf_e/Isatidis_Radix_v4_e.pdf). Half gram of BLG sample powder was weighed and poured into a 50 mL tube loaded with 20 mL of water. The tubes were then placed in a boiling water bath for a one-hour extraction. The tubes were then centrifuged (at 1,800 x g) for 0.25 hour. The resultant supernatant was moved to a 25 mL volumetric flask. The residue was rinsed with water and combined with the collected supernatant. The volumetric flask was filled up to the graduation mark with water. The extracts were than filtered through 0.45 μm PTFE filters. Ten microliters of the water extract of each sample were then injected into the Vanquish Flex UHPLC System (Thermo Scientific, USA) by an autosampler and eluted by a gradient program of 0–10 min A = 98%: B = 2%, 10–40 min A = 98% to 85%: B = 2% to 15%, 40–60 min A = 85% to 98%: B = 15% to 2%, where A is 0.02% phosphoric acid in water and B is methanol, at a flow rate of 0.6 mL/min. The dimension of the chromatographic column (Shimadzu C_18_, Kyoto, Japan) was 4.6 mm x 25 cm with 5 μm particle size, and the temperature was controlled at 35°C. An ultraviolet wavelength of 240 nm was applied for the detection of epigoitrin. Several concentrations of epigoitrin chemical standard (Chengdu Alfa, Sichuan, China) were injected into the instrument three times to set a curve for the comparison of epigoitrin content in samples. A standard BLG obtained from National Institutes for Food and Drug Control was extracted and chromatographed in parallel with samples for comparison. The standard requirement of epigoitrin was not less than 0.020% in the Chinese Pharmacopoeia and not less than 0.029% in the HKCMMS based on the same extract concentration of the water extracts. The quantification was performed with Thermo Scientific Chromeleon 7 software.

#### Indirubin extraction from Daqingye and HPLC assay.

DQY samples were extracted according to the HPLC fingerprinting method in HKCMMS (https://www.cmro.gov.hk/files/hkcmms/vol4/pdf_e/Isatidis_Folium_v4_e.pdf). Six hundred milligrams of sample powder were weighed and transferred to a 50 mL capped tube, followed by the addition of 40 mL ethyl acetate for sonication extraction for one hour. The solution was then centrifuged (at 3,000 x g), and the supernatant was obtained and dried, followed by an addition of 25 mL of methanol for the resuspension. The resultant solution was than filtered through a 0.45 PTFE filter. Twenty microliters of each filtered sample extract were injected into the Vanquish Flex UHPLC System (Thermo Scientific, USA) equipped with the Shimadzu C_18_ column (4.6 mm x 25 cm, 5 μm). The chromatogram was detected at ultraviolet wavelengths of 250 nm and 291 nm. The elution program of the chromatography was based on the HKCMMS, as 0–10 min A = 80% to 75%: B = 20% to 22%, 10–20 min A = 78% to 45%: B = 22% to 55%, 20–45 min A = 45% to 53%: B = 55% to 57%, 45–60 min A = 43% to 40%: B = 57% to 60%, where A is water and B is acetonitrile, at a flow rate of 1 mL/min. The column temperature was maintained at 40 °C. Different concentrations of indirubin were injected three times to set a standard curve for quantifying the amount of indirubin in the samples. The samples should contain no less than 0.020% of indirubin for meeting the listed requirement. The results were analyzed with the Thermo Scientific Chromeleon 7 software.

## Results

### Species identification

#### Morphological authentication of Banlangen samples.

Using the Chinese Pharmacopoeia as a reference, we found that only one sample matched the expected morphology of the genuine species (S1 Fig). Initially, we were uncertain about the identities of the vast majority of samples. After conducting DNA barcoding, we discovered that most samples belonged to the *Strobilanthes* genus (S2 Fig). We then verified that the morphological descriptions aligned with the identities revealed by the DNA analysis (S1 File).

#### DNA authentication results of Banlangen samples.

DNA barcoding at the *ITS2* region showed only the sample T5569 obtained from Shatin matched the expected species identity of *Isatis tinctoria* (Banlangen). T5562 obtained from Sai Kung was a mixture of species *Strobilanthes* and *Dictamnus dasycarpus* Turcz. as known as Baixianpi in Chinese. The other BLG samples were all *Strobilanthes* species. The DNA sequences for the *rbcL* region confirmed the species identities. The found identities of Banlangen samples are shown in Table 2. The sequences are presented in S3 File.

**Table 2:** Authentication results of Banlangen samples by DNA barcoding.

		*ITS2* region	*rbcL* region
#	Code	Species; Accession no. in GenBank: Coverage, Similarity	
1	T5557	*Strobilanthes hamiltoniana*^; KT004497.1: 87%, 98.10%	*Strobilanthes dimorphotricha*; KJ939249.1: 100%,100%
		*Strobilanthes species*; MT896162.1: 100%, 96.98%	*Strobilanthes bantonensis*; MT576695.1; 100%, 99.80%
		*Strobilanthes anamallaica*; MT876221.1: 100%, 96.98%	*Strobilanthes cusia*; NC_037485.1: 100%, 99.60%
		*Strobilanthes ciliate*; MT876220.1: 100%, 96.98%	
2	T5558	*Strobilanthes hamiltoniana*; KT004497.1: 100%, 99.43%	(Not available)
3	T5559	*Strobilanthes hamiltoniana*^; KT004497.1: 93%, 98.10%	(Not available)
		*Strobilanthes species*; MT896162.1: 100%, 96.79%	
		*Strobilanthes anamallaica*; MT876221.1: 100%, 96.79%	
		*Strobilanthes ciliate*; MT876220.1: 100%, 96.79%	
4	T5560	*Strobilanthes hamiltoniana*; KT004497.1: 100%, 99.43%	*Strobilanthes bantonensis*; MT576695.1: 100%, 100%
			*Strobilanthes hamiltoniana*; MZ269002.1: 100%, 99.80%
			*Strobilanthes yunnanensis*; MF786508.1: 100%, 99.80%
5	T5561	(Not available)	*Strobilanthes dimorphotricha*; KJ939249.1: 100%, 100%
			*Strobilanthes bantonensis*; MT576695.1; 100%, 99.80%
			*Strobilanthes cusia*; NC_037485.1: 100%, 99.80%
6	T5562	(Not available)	*Strobilanthes crispa*; MH069849.1: 100%, 100%
			*Strobilanthes oresbia*; MH185942.1: 100%, 100%
			*Strobilanthes cusia*; NC_037485.1: 100%, 100%
		*Dictamnus species*; FJ593176.1; 100%, 100%	(Not available)
		*Dictamnus albus*; MH711176.1; 100%, 99.42%	
		*Dictamnus angustifolius*; MT924084.1; 100%, 99.42%	
		*Dictamnus dasycarpus*; MW264240.1; 88%, 100%	
7	T5563	*Strobilanthes hamiltoniana*; KT004497.1: 100%, 99.43%	*Strobilanthes bantonensis*; MT576695.1; 100%, 100%
			*Strobilanthes cusia*; NC_037485.1: 100%, 100%
			*Strobilanthes yunnanensis*; MF786508.1: 100%, 99.80%
8	T5564	(Not available)	*Strobilanthes bantonensis*; MT576695.1: 100%, 100%
			*Strobilanthes cusia*; NC_037485.1: 100%, 100%
			*Strobilanthes yunnanensis*; MF786508.1: 100%, 99.80%
9	T5565	*Strobilanthes hamiltoniana*^; KT004497.1: 94%, 98.36%	(Not available)
		*Strobilanthes species*; MT896162.1: 100%, 95.65%	
		*Strobilanthes anamallaica*; MT876221.1: 100%, 95.65%	
		*Strobilanthes ciliate*; MT876220.1: 100%, 95.65%	
10	T5566	*Strobilanthes hamiltoniana*; KT004497.1: 100%, 99.43%	*Strobilanthes bantonensis*; MT576695.1; 100%, 100%
			*Strobilanthes cusia*; NC_037485.1: 100%, 99.80%
			*Strobilanthes yunnanensis*; MF786508.1: 100%, 99.80%
11	T5567	*Strobilanthes hamiltoniana*^; KT004497.1: 87%, 98.10%	*Strobilanthes bantonensis*; MT576695.1; 100%, 100%
		*Strobilanthes anamallaica*; MT876221.1: 100%, 97.32%	*Strobilanthes cusia*; NC_037485.1: 100%, 99.80%
		*Strobilanthes ciliate*; MT876220.1: 100%, 97.32%	*Strobilanthes yunnanensis*; MF786508.1: 100%, 99.80%
12	T5568	*Strobilanthes hamiltoniana*^; KT004497.1: 87%, 98.10%	*Strobilanthes bantonensis*; MT576695.1: 100%, 100%
		*Strobilanthes species*; MT896162.1: 100%, 96.98%	*Strobilanthes cusia*; NC_037485.1: 100%, 99.80%
		*Strobilanthes anamallaica*; MT876221.1: 100%, 96.98%	*Strobilanthes yunnanensis*; MF786508.1: 100%, 99.80%
		*Strobilanthes ciliate*; MT876220.1: 100%, 96.98%	
13	T5569	*Isatis tinctoria*; FJ593182.1: 100%, 100%	(Not available)
		*Isatis indigotica*; AF384104.1: 100%, 100%	
14	T5570	*Strobilanthes hamiltoniana*; KT004497.1: 98%, 100%	*Strobilanthes bantonensis*; MT576695.1; 100%, 100%
		*Strobilanthes hamiltoniana*; MT845223.1: 98%, 98.98%	*Strobilanthes cusia*; NC_037485.1: 100%, 99.80%
		*Strobilanthes kunthiana*; OK483360.1: 95%, 97.89%	*Strobilanthes yunnanensis*; MF786508.1: 100%, 99.80%
15	T5571	*Strobilanthes hamiltoniana*^; KT004497.1: 87%, 98.10%	(Not available)
		*Strobilanthes anamallaica*; MT876221.1: 100%, 96.98%	
		*Strobilanthes ciliate*; MT876220.1: 100%, 96.98%	
16	T5572	*Strobilanthes hamiltoniana*^; KT004497.1: 87%, 98.48%	*Strobilanthes bantonensis*; MT576695.1: 100%, 100%
		*Strobilanthes anamallaica*; MT876221.1: 100%, 97.32%	*Strobilanthes cusia*; NC_037485.1: 100%, 99.80%
		*Strobilanthes ciliate*; MT876220.1: 100%, 97.32%	*Strobilanthes yunnanensis*; MF786508.1: 100%, 99.80%
17	T5573	(Not available)	*Strobilanthes dimorphotricha*; KJ939249.1: 100%, 100%
			*Strobilanthes bantonensis*; MT576695.1; 100%, 99.80%
			*Strobilanthes cusia*; NC_037485.1: 100%, 99.80%
18	T5574	(Not available)	*Strobilanthes bantonensis*; MT576695.1: 100%, 100%
19	T5624	(Not available)	*Strobilanthes bantonensis*; MT576695.1: 100%, 100%
			*Strobilanthes cusia*; NC_037485.1: 100%, 99.80%
			*Strobilanthes yunnanensis*; MF786508.1: 100%, 99.80%

**Note:** ^The blast results for *Strobilanthes* species showed the highest similarity at > 98%, but with a lower coverage of 94%. This reduced coverage is attributed to our sequences being longer than the available reference sequences in GenBank.

#### Morphological authentication results of Daqingye samples.

Using the Chinese Pharmacopoeia as a reference, we found only one sample that matched the expected morphology of the genuine species, *Isatis tinctoria* (S3 Fig). DNA barcoding revealed that the majority belonged to the *Strobilanthes* genus, with one sample identified as a *Blumea* species. We then confirmed that the morphological descriptions aligned with the DNA analysis results. In summary, we identified three morphological patterns (S3–5 Figs).

#### DNA authentication results of Daqingye samples.

The species identities of samples were deduced at the highest similarity by the highest scores in blastN organism taxonomy hits and alignment results. Only the sample T5543 matched the expected identity of *Isatis tinctoria* (Daqingye) in the Chinese Pharmacopoeia at the *ITS2*, *rbcL*, *matK* and *psbA-trnH* regions. Sample T5537 was adulterated by *Blumea balsamifera* (L.) DC. as revealed at the *ITS2* and *rbcL* regions. Other samples were identified to be *Strobilanthes* species. Fifteen samples contained *Strobilanthes cusia* and eight samples contained *Strobilanthes dimorphotricha*, identified by the *matK* and *psbA-trnH* regions. Sequencing results of *Strobilanthes* species and *Isatis* species were not ideal at the *ITS2* region due to plant-fungal double peaks [23,24].

A summary of the results is presented as in Table 3. The sequences are in S4 File.

**Table 3:** Authentication results of Daqingye samples by DNA barcoding.

	*ITS2* region	*rbcL* region	*psbA-trnH* region	*matK* region
**Code**	**Species; Accession no. in GenBank: Coverage, Similarity**			
T5529	(Not available)	*Strobilanthes dimorphotricha;* KJ939249.1: 100%, 100%	*Strobilanthes cusia*^#^; NC_037485.1 *Baphicacanthus cusia;* KT161373.1: 100%, 100%	*Strobilanthes cusia*^#^; NC_037485.1: 100%, 99.63% *Baphicacanthus cusia;* KJ939222.1: 100%, 99.63%
T5530	(Not available)	*Strobilanthes dimorphotricha;* KJ939249.1: 100%, 99.80%	*Strobilanthes* species *Baphicacanthus cusia;* KT161373.1: 100%, 100%; *Strobilanthes thwaitesii;* OP320814.1: 100%, 100%; *Strobilanthes lupulina;* OP320807.1: 100%, 100%; among others	(Not available)
T5531	(Not available)	*Strobilanthes species* *Strobilanthes cusia;* NC_037485.1: 100%, 100%; *Strobilanthes yunnanensis;* MF786508.1: 100%, 100%; *Strobilanthes hamiltoniana;* MZ269002.1: 100%, 100%; among others	*Strobilanthes species* *Baphicacanthus cusia;* KT161373.1: 100%, 100%; *Strobilanthes hamiltoniana* KT161354.1: 100%, 100%; *Strobilanthes thwaitesii;* OP320814.1: 100%, 100%; among others	(Not available)
T5532	(Not available)	*Strobilanthes dimorphotricha;* KJ939249.1: 100%, 100%	*Baphicacanthus cusia;* KT161373.1: 100%, 100%	*Baphicacanthus cusia;* KJ939222.1: 100%, 99.63%
T5533	(Not available)	*Strobilanthes dimorphotricha;* KJ939249.1: 100%, 100%	*Baphicacanthus cusia;* KT161373.1: 100%, 100%	(Not available)
T5535	(Not available)	*Strobilanthes dimorphotricha;* KJ939249.1: 100%, 100%	*Baphicacanthus cusia;* KT161373.1: 100%, 100%	(Not available)
T5536	(Not available)	*Strobilanthes dimorphotricha;* KJ939249.1: 100%, 100%	*Baphicacanthus cusia*; KT161373.1: 100%, 100%	*Strobilanthes dimorphotricha*^; KP093335.1: 95%, 100%
T5537	*Blumea balsamifera*; KX534370.1: 100%, 99.75%	*Blumea balsamifera;* MH069751.1: 100%, 100%	(Not available)	(Not available)
T5538	(Not available)	*Strobilanthes dimorphotricha;* KJ939249.1: 100%, 100%	*Baphicacanthus cusia;* KT161373.1: 100%, 100%	*Strobilanthes dimorphotricha*^; KP093335.1: 92%, 100%
T5539	(Not available)	*Strobilanthes dimorphotricha;* KJ939249.1: 100%, 100%	*Baphicacanthus cusia;* KT161373.1: 100%, 100%	(Not available)
T5540	(Not available)	(Not available)	*Baphicacanthus cusia;* KT161373.1: 100%, 100%	*Strobilanthes dimorphotricha*^; KP093335.1: 95%, 100%
T5541	(Not available)	(Not available)	*Baphicacanthus cusia;* KT161373.1: 100%, 100%	*Strobilanthes dimorphotricha*^; KP093335.1: 95%, 100%
T5542	(Not available)	*Strobilanthes dimorphotricha;* KJ939249.1: 100%, 100%	*Baphicacanthus cusia;* KT161373.1: 100%, 100%	(Not available)
T5543	*Isatis tinctoria*; JF421508.1: 100%, 100%	*Isatis tinctoria*; OL827242.1: 100%, 100%; *Isatis violascens*; OQ644473.1: 100%, 100%; *Isatis gymnocarpa*; OQ644470.1: 100%, 100%	*Isatis tinctoria*; QR936036.1: 100%, 100%	*Isatis tinctoria*; MK435776.1: 100%, 100%
T5544	(Not available)	*Strobilanthes dimorphotricha;* KJ939249.1: 100%, 99.80%	*Baphicacanthus cusia;* KT161373.1: 100%, 100%	*Strobilanthes dimorphotricha*^; KP093335.1: 95%, 100%
T5547	(Not available)	*Strobilanthes species* *Strobilanthes oresbia;* MH185942.1: 100%, 100%; *Strobilanthes gossypina;* MF349322.1: 100%, 100%; *Strobilanthes dalzielii:* NC_084379.1: 100%, 100%; among others	*Strobilanthes species* *Strobilanthes dalzielii;* NC_084379.1: 100%, 100%; *Strobilanthes sexennis;* OP320813.1: 100%, 100%; *Strobilanthes pulcherrima;* OP320808.1: 100%, 100%; *Strobilanthes habracanthoides;* OP320801.1: 100%, 100%; *Strobilanthes biocullata;* NC_060347.1: 100%, 100%;	*Strobilanthes dalzielii;* NC_084379.1: 100%, 99.88%
T5549	(Not available)	*Strobilanthes dimorphotricha;* KJ939249.1: 100%, 100%	*Baphicacanthus cusia;* KT161373.1: 100%, 100%	*Strobilanthes dimorphotricha*^; KP093335.1: 95%, 100%
T5550	(Not available)	*Strobilanthes species* *Strobilanthes cusia;* NC_037485.1: 100%, 100%; *Strobilanthes yunnanensis;* MF786508.1: 100%, 100%; *Strobilanthes hamiltoniana;* MZ269002.1: 100%, 100%; among others	*Baphicacanthus cusia;* KT161373.1; 100%, 100%	*Baphicacanthus cusia;* KJ939222.1: 100%, 99.35%
T5551	(Not available)	(Not available)	*Baphicacanthus cusia;* KT161373.1; 100%, 100%	*Strobilanthes dimorphotricha*^; KP093335.1: 95%, 100%

Note:

# *Strobilanthes* and *Baphicacanthus* are synonymous. ^The blast results for *Strobilanthes* species showed the highest similarity at > 98%, but with a lower coverage of 94%. This reduced coverage is attributed to our sequences being longer than the available reference sequences in GenBank.

## HPLC results

### HPLC quantification of Epigoitrin in Banlangen

**Fig 1:**
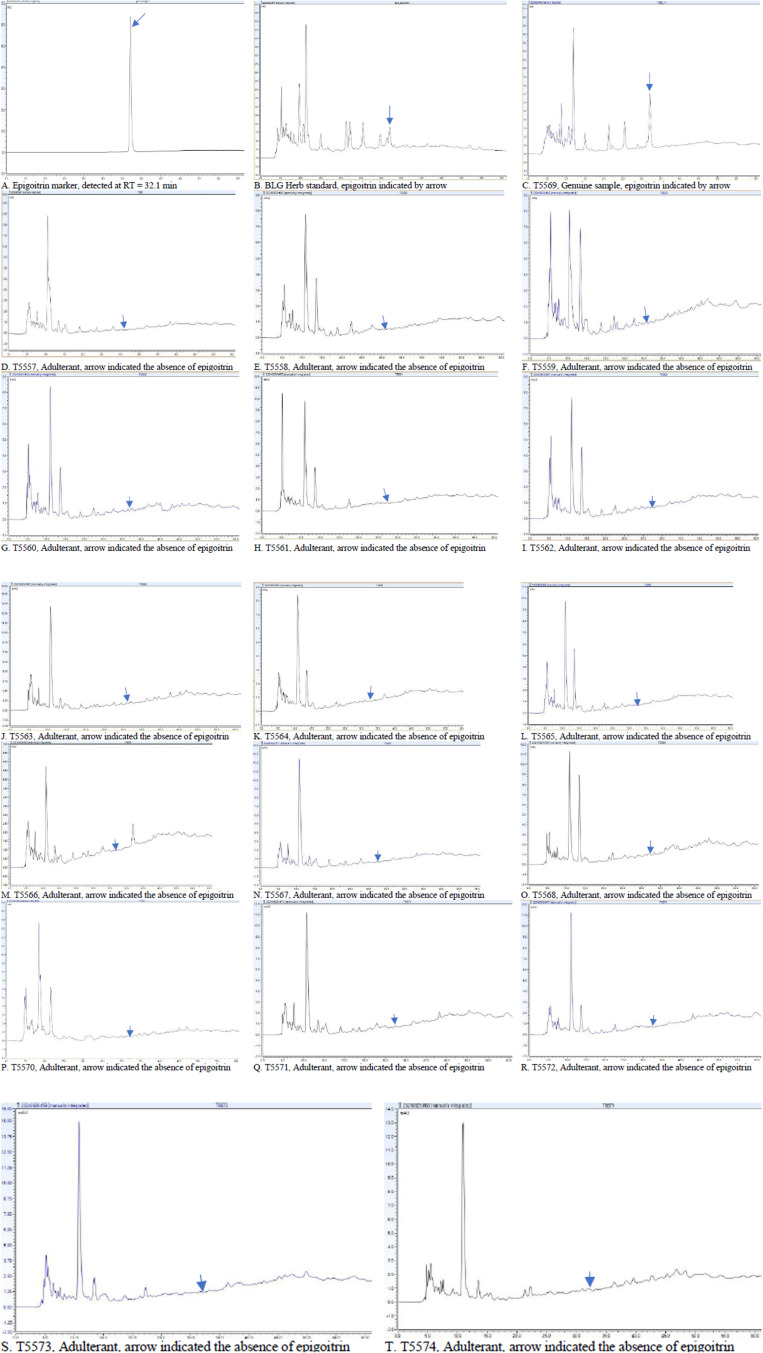
HPLC profiles of Banlangen samples. Arrow indicates the retention timepoint of the marker epigoitrin.

By comparison with the established standard curve y = 1.8824x - 0.4489,

R² = 1 (S6 Fig in S5 File), only the sample T5569 that was identified as genuine Banlangen species contained 0.023% of epigoitrin, meeting the standard requirement in the Chinese Pharmacopoeia (>0.020%) but failed that in the HKCMMS (<0.029%). The chromatograms of samples obtained from other seventeen districts did not show the peak of epigoitrin and the chromatographic profiles were different from that of the genuine BLG sample. Fig 1A displays the chromatogram of the epigoitrin marker. Fig 1B presents the chromatogram of the BLG herb standard. Fig 1C shows the chromatogram of the genuine sample T5569. Figs 1D–T illustrate other samples containing *Strobilanthes* adulterant species. The BLG herb standard was used as a reference. The chromatograms demonstrated consistency, as both the genuine sample and the herb standard contained epigoitrin.

### HPLC quantification of indirubin in Daqingye

**Fig 2:**
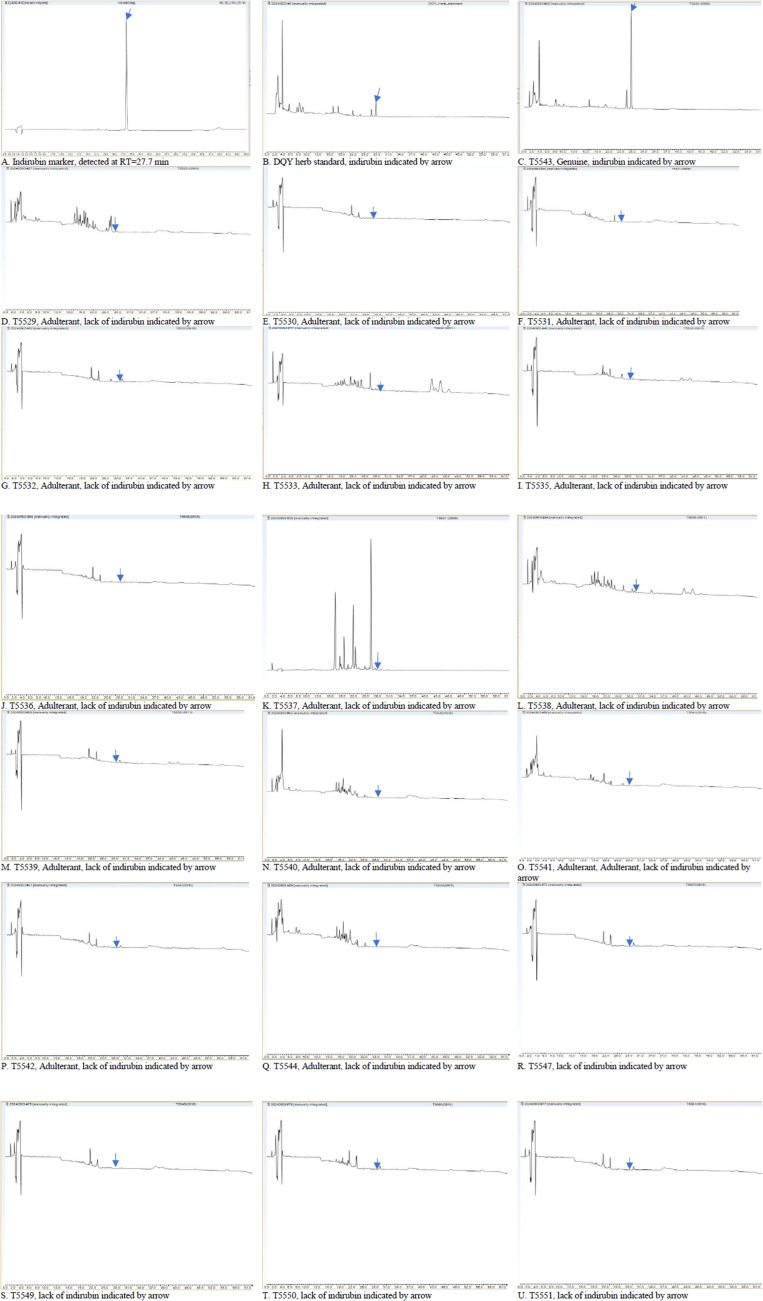
HPLC profiles of Daqingye samples. Arrow indicates the retention timepoint of the marker indirubin.

By comparison with the established standard curve y = 1.6525x + 0.2153, R² = 0.9996 (S7 Fig in S6 File), only the sample T5543 that was identified as genuine Daqingye sample met the standard requirement of indirubin at 0.020% (Fig 2C). Fig 2A shows the chromatogram of indirubin. Fig 2B displays the chromatograph of DQY herb standard. Figs 2D-U illustrate the chromatograms of the substituted samples. The *Strobilanthes* species (Figs 2D-J; L-U) and *Blumea balsamifera* (Fig 2K) exhibited distinct profiles compared to those of the DQY herb standard and the genuine sample.

## Discussion

### Incorrect species

In this work, we discovered the detected species of BLG and DQY in Hong Kong market was not the same as that in the official standard, that only one sample obtained from the eighteen districts was genuine for both herbs, as found by our molecular and chemical analyses. A shop sold both genuine and adulterant herbs and provided both to us in separate parcels, as the staff had the knowledge to differentiate between the two herbs. It was unacceptable for these frequently prescribed herbs to be of wrong identities. Most adulteration was due to the substitution by the *Strobilanthes* species, an adulterant species that has been reported by the Department of Health [25]. A sample of DQY was completely adulterated by *Blumea balsamifera*, a rarely reported adulterant species. The chemical profiles of wrong species were different from that of the genuine species in our findings, that the expected bioactive marker was only detectable in the genuine species but not the wrong species. This showed that the chemical content of the substituting species was distinct from the genuine species, and could result in completely different treatment effects and health consequences. In particular, *Blumea balsamifera* is effective for lipid lowering, wound healing and pain relief. Besides, it can be toxic to the certain liver and cancer cells. Incorrect species can thus lead to safety issues in addition to quality issues [26].

Species substitution was not limited to DQY but was also a widespread issue in Hong Kong and elsewhere. Qianliguang (*Senecio scandens* Buch. -Ham. ex D. Don) has been found to be commonly substituted by *Lespedeza cuneata* (Dum.Cours.) G.Don and *Achyranthes aspera* L., with the authentic samples tested to be overly toxic [27]. Five Flowers Tea, a cool beverage containing five herbs, was discovered to include impurities, substitutes, fillers, insects, and ten unexpected species [28]. Worldwide, 27% of the commercial herbal products were found to be adulterated [29]. This study, combined with the results of previous research, demonstrates an urgent need to address the global and regional herb substitution to ensure the efficacy and safety of medicinal herbs and uphold the reputation of the Chinese medicine industry.

### The substitution with *Strobilanthes* species

The substitution of genuine BLG and DQY (*Isatis tinctoria*) with NanBLG and NanDQY (*Strobilanthes* species) [30] may be attributed to preferences within the Chinese medicine industry in Hong Kong. Several electronic information databases, provided to Chinese medicine practitioners and monitored by licensed companies under the Hong Kong Science and Technology Parks Corporation’s incubation programme, indicate that *Strobilanthes cusia* is used as an alternative to *Isatis tinctoria* [31]. This difference is related to the greater availability of this herb in southern China, in contrast to the genuine species, which is more prevalent in northern China [2].

Traditionally, root and leaf parts of *Isatis* [32] and *Strobilanthes* [33] species can both be used to treat influenza. Researchers compared the flu inhibition by NanBLG (root of *Strobilanthes* species) and BLG (root of *Isatis* species) and found that they both had significant anti-influenza activity [33]. BLG was more effective in direct virus inhibition and prevention of the virus infection, while NanBLG (root of *Strobilanthes* species) was more effective to cure the virus after the infection.

Extracted compounds Strobilanthes A (1) and 2(3H)-benzoxazolinone from Nandaqingye (leaf of *Strobilanthes* species) also showed potent anti-influenza activity [34]. Despite *Isatis* and *Strobilanthes* species both gave anti-influenza effects, we found that the chemicals of *Strobilanthes* species and *Isatis* species were very different by comparison of the chromtographic profiles. Our findings showed *Strobilanthes* species (NanBLG and NanDQY) did not give the peaks of chemical standard markers epigoitrin and indirubin of BLG or DQY (*Isatis* genus) respectively.

The Department of Health in Hong Kong has already provided educational materials showing *Isatis* and *Strobilanthes* species have distinct functions and should not be interchangeably used.

### Other adulteration of species

Apart from the adulteration of *Strobilanthes* species, we found *Blumea balsamifera* (Ainaxiang) was a substituting species of one Daqingye sample, and *Dictamnus dasycarpus* (Baixianpi) was mixed with Nanbanlangen in one sample. These revealed inaccuracy in the supply chain practices that resulted in wrongly dispensed herbs. Baixianpi, unlike Nanbanlangen that treats flu symptoms, is commonly used for treating moisture in skin disorder such as eczema [35]. The appearance of Baixianpi and Nanbanlangen are different despite their white color [35]. Baixianpi pieces are curl in shape and without woody core [35], but Nanbanlangen has larger pieces and has pith in the center [19]. The mixture found in the sample showed the carelessness in storage and dispensing of herbs with same color, even when the two herbs have different morphology.

The adulterant Ainaxiang is a distinct plant and has different morphology and indication from Daqingye. Unlike Daqingye, it is indicated for treating respiratory symptoms, arthritis pain, headache and menarche, and is not known for virus inhibition activity [36]. The herb handlers might have stored the sample in the shop with wrong labelling, and then dispensed the wrong herbs.

### Comparing the outcomes of DNA barcoding and chemical analysis

The *rbcL* region was not recommended for the species authentication of *Strobilanthes* species (NanBLG and NanDQY) as the region had low evolutionary rate and resulted in the insufficiency to differentiate among closely related species. The sequencing results at the *ITS2* region were non-ideal due to co-amplification of fungal sequences, a characteristic of this region [23,24]. *psbA-trnH* and *matK* were more suitable for the authentication of *Strobilanthes* species. Nonetheless, more than one species were possible for several samples across three DNA regions, indicating that the references available in GenBank might be insufficient for accurate species identification within the *Strobilanthes* genus. DNA barcoding was more effective in identifying the authentic species of *Isatis* (BLG and DQY). It is recommended that future research adopt simultaneous multi-locus DNA authentication and upload the resulting sequences to the database, particularly for *Strobilanthes* species. Enhancing the available sequences in the database will enable researchers to more accurately determine species classifications. Chemical analyses also revealed that only one sample of BLG and one sample of DQY was *Isatis tinctoria*. The substituted samples consistently lacked the marker peak of the genuine species. The adoption of standardized protocols in chemical analyses has facilitated universal application and rapid screening. Our findings indicate that chemical analysis should be conducted alongside DNA barcoding for the authentication of BLG and DQY.

### Future implications

Our work found that only one sample each obtained from eighteen shops was genuine which was unacceptable for both DQY and BLG in Hong Kong. The serious adulteration or substitution can produce different treatment effects owing to the differences in the chemical composition. Track-and-trace system such as the HerBChain [37] for the control of the source of the herbs and supply chain practices is suggested to be implemented. It is recommended to educate the herbalists to source the correct herbs or label the herbs properly, for consumers to be aware of the identity of the herb they obtained. This is important as the adulterated species does not contain the same chemical content as the genuine herbs. We suspect the substitution of *Isatis tinctoria* by *Strobilanthes* species happens not only in Hong Kong but also worldwide, as Hong Kong is a major entrepôt of herbal material to overseas countries.

## Conclusion

BLG and DQY have a long history of being utilized for a wide range of applications, including sustaining immunity, fighting against viruses, reducing inflammation, mitigating oxidation, and relieving lung heat. This work explores the quality and authenticity of BLG and DQY (*Isatis tinctoria*) sold in the Hong Kong market. We discovered serious substitution of genuine DQY by *Strobilanthes* species. Out of 18 samples of BLG, 17 samples were identified as *Strobilanthes* species, with one sample containing pieces of Baixianpi (*Dictamnus dasycarpus*). Only one sample was found to be genuine. Among 19 samples of DQY, 16 samples were found to be *Strobilanthes* species, and one sample was adulterated with Ainaxiang (*Blumea balsamifera*). Again, only one sample was genuine. The issue of substitution revealed mislabeling issues in herb shops. This poses a public health risk, highlighting the need for improved quality control of these two popular herbs to maximize their medicinal benefits. Additionally, potential toxicity from substituted herbs can cause irreversible harm. This study underscores the urgent need to address these errors.

## Supporting information

S1 File Organoleptic characteristics and morphological identities of samples. (DOCX)

S2 File PCR amplification protocols. (DOCX)

S3 File DNA sequences of Banlangen samples. (DOCX)

S4 File DNA sequences of Daqingye samples. (DOCX)

S5 File Calibration curve of epigoitrin by HPLC method. (DOCX)

S6 File Calibration curve of indirubin by HPLC method. (DOCX)
