# Supplementary material for: Quality assessment of medicinal material Daqingye and Banlangen from Isatis tinctoria Fort. reveals widespread substitution with Strobilanthes species
Source: PLoS One. 2025 May 7;20(5):e0323084. doi: 10.1371/journal.pone.0323084 (PMC12058189; doi:10.1371/journal.pone.0323084)
Supplement: S1 File — (DOCX) [file pone.0323084.s001.docx]

**S1 File. Organoleptic characteristics and morphological identities of samples**

**1a. Images of samples of Banlangen**


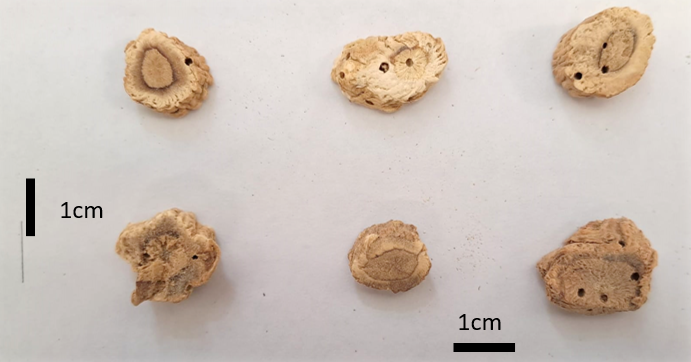


**S1 Fig. A photo of genuine *Isatis* species.** Note: Banlangen is cylindrical in shape, with a pale gray-yellow or light brown-yellow surface. It has longitudinal wrinkles, horizontal elongated pore-like protrusions, and traces of supporting roots. It is solid in texture, slightly soft, with a yellow-white skin section and a yellow wood section. Sample T5569 is genuine *Isatis* species.


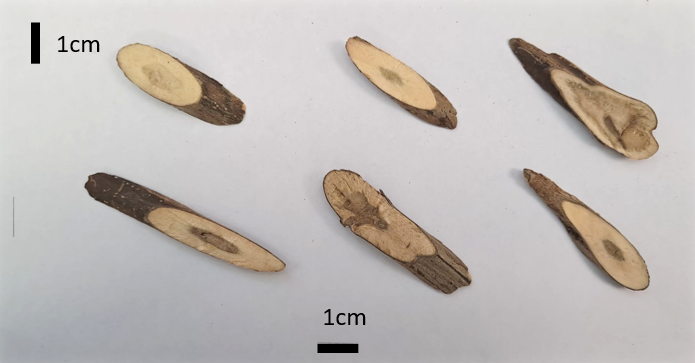


**S2 Fig. A photo of substituted Southern (*Strobilanthes*) species**. Note: Substituted *Strobilanthes* species are with pith in the center and exterior skin in grayish color. The texture is hard and brittle, easily broken, with an uneven fracture surface. Samples T5557-T5568 and T5570-T5574 are *Strobilanthes* species.

**1b. Images of samples of Daqingye**


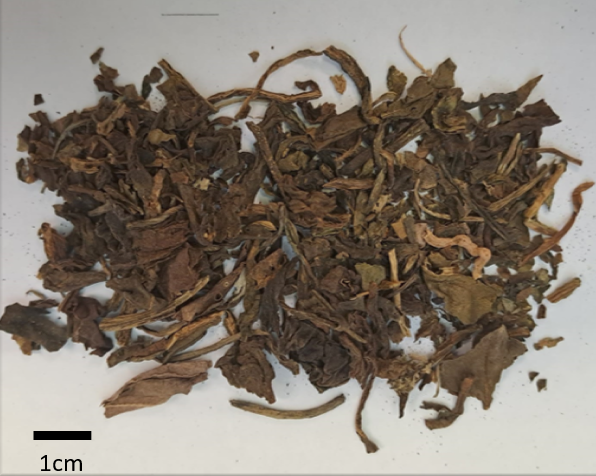


**S3 Fig. A photo of genuine *Isatis* species.** Note: The leaves are often wrinkled and curled, with some fragments present. When flattened, they take on an elongated oval shape, resembling an inverted lanceolate form. The upper surface is dark gray-green, featuring slightly raised, darker dots. The tip is blunt, with a smooth or slightly wavy margin, and the base narrows to form a wing-like shape, appearing light brownish-yellow. The texture is brittle, with a faint aroma and a taste that is slightly sour, bitter, and astringent.


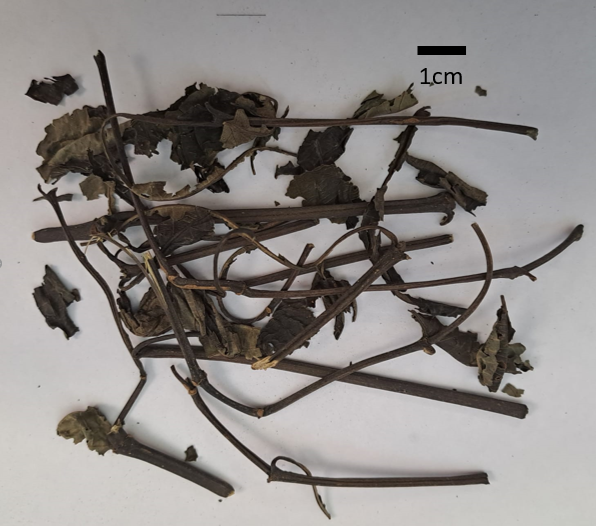


**S4 Fig. A photo of substituted *Strobilanthes* species.** Note: The leaf is primarily broken and features an acuminate apex when flattened. It has a blackish-green to greyish-green blade with small obtuse serrations along the margin. The midrib is notably prominent on the dorsal side, leading to a cuneate decurrent base. The petiole is also blackish-green or greyish-green, and the branchlet is sub-square with a similar surface coloration.


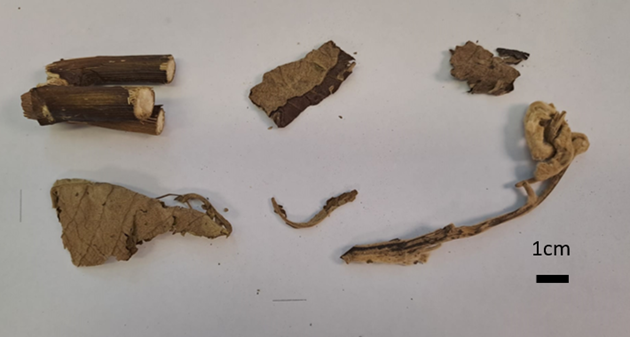


**S5 Fig. A photo of substituted *Blumea* species.** Note: The stem of this plant is cylindrical, branched, and has a surface that is gray-green or gray-brown, featuring shallow longitudinal ridges covered in grayish-white hairs. It is brittle and easily breaks, with a white pith at the center of the broken surface. The leaves are alternate, wrinkled or fragmented; when flattened, the complete leaves appear oval or elongated lanceolate, with irregular upward-curving serrations along the edges. The leaf surface is yellow-green or gray-green, slightly wrinkled and somewhat rough, covered in short hairs, while the underside is densely covered in white or light brownish-yellow hairs. The leaves are slightly flexible and brittle when dry. They have a pleasant aroma and a bitter, pungent taste.
