# Supplementary material for: Quality assessment of medicinal material Daqingye and Banlangen from Isatis tinctoria Fort. reveals widespread substitution with Strobilanthes species
Source: PLoS One. 2025 May 7;20(5):e0323084. doi: 10.1371/journal.pone.0323084 (PMC12058189; doi:10.1371/journal.pone.0323084)
Supplement: S2 File — (DOCX) [file pone.0323084.s002.docx]

**S2 File. PCR amplification protocols**

| **Primer** | **Primer name** | **Primer sequence (5’ to 3’)** | **PCR protocol** | **DNA locus** |
| --- | --- | --- | --- | --- |
| rbcL1/rbcLB | rbcL1  rbcLB | TTG GCA GCA TTY CGA GTA ACT CC  AAC CYT CTT CAA AAA GGT C | Initial denaturation:  95 °C for 2.5 min  10 cycles of  [30 s at 95 °C denaturation, 30 s at 52 °C annealing,  30 s at 72 °C extension]  25 cycles of  [30 s at 88 °C denaturation, 30 s at 52 °C annealing, 30 s at 72 °C extension]  Final extension:  60 °C for 10 min  Enzyme deactivation:  4 °C for 5 min | *rbcL* |
| rbcLa_F/rbcLa_R | rbcLa_F  rbcLa_R | ATGTCACCACAAACAGAGACTAAAGC  GTAAAATCAAGTCCACCRCG | Initial denaturation:  95 °C for 4 min  35 cycles of  [30 s at 94 °C denaturation,  1 min at 55 °C annealing,  1 min at 72 °C extension]  Final extension:  72 °C for 10 min | *rbcL* |
| 3F-KIM/1R-KIM | 3F-KIM  1R-KIM | CGTACAGTACTTTTGTGTTTACGAG  ACCCAGTCCATCTGGAAATCTTGGTTC | Initial denaturation:  95 °C for 2 min  45 cycles of  [30 s at 95 °C denaturation, 30 s at 50 °C annealing, 40s at 72 °C extension]  Final extension:  72 °C for 5 min  Enzyme deactivation:  4 °C for 5 min | *matK* |
| ITS2F/ITS3R | ITS-S2F  ITS-S3R | ATG CGA TAC TTG GTG TGA AT  GAC GCT TCT CCA GAC TAC AAT | Initial denaturation:  94°C for 5 min  40 cycles of  [30 s at 94°C denaturation, 30 s at 56°C annealing, and 45 s at 72°C extension]  Final extension:  72°C for 10 min  Enzyme deactivation:  4 °C for 5 min | *ITS2* |
| ITSu3/ITSu4  ITSp3/ITSu4 | ITSp3  ITSu3  ITSu4 | YGA CTC TCG GCA ACG GAT A  CAW CGA TGA AGA ACG YAG C  RGT TTC TTT TCC TCC GCT TA | Initial Denaturation:  94 °C for 4 min  40 cycles of  [30 s at 94 °C denaturation, 40 s at 50 °C annealing, 1 min at 72 °C extension]  Final extension:  72 °C for 10 min  Enzyme deactivation:  4 °C for 5 min |  |
| psbAF/trnHR | psbAF  trnHR | GTT ATG CAT GAA CGT AAT GCT C  CGC GCA TGG TGG ATT CAC AAT CC | Initial Denaturation:  94 °C for 5 min  30 cycles of  [1 min at 94 °C denaturation, 1 min at 56 °C annealing, 1.5 min at 72 °C extension]  Final extension:  72 °C for 7 min  Enzyme deactivation:  4 °C for 5 min | *psbA-trnH* |
