# Supplementary material for: Quality assessment of medicinal material Daqingye and Banlangen from Isatis tinctoria Fort. reveals widespread substitution with Strobilanthes species
Source: PLoS One. 2025 May 7;20(5):e0323084. doi: 10.1371/journal.pone.0323084 (PMC12058189; doi:10.1371/journal.pone.0323084)
Supplement: S3 File — (DOCX) [file pone.0323084.s003.docx]

**S3 File. DNA sequences of Banlangen samples**

**3a. DNA Sequences of Banlangen samples at the *ITS2* region (primers ITSp3/ITSu4)**

>T5557

TGCGATACTTGGTGTGAATTGCAGAATCCCGTGAACCATCGAGTCTTTGAACGCAAGTTGCGCCCGAAGCCTTCGGGCCGAGGGCACGCCTGCCTGGGCGTCACGCATCGCGTCGCCCCCCCTACCCCGCTCGAACGGAGCGGGTGCGGCGGGGGCGGATGTTGGCCTCCCGTGCGTCCCCGTGCGGTTGGCCCAAATTGGATCCCCCGGCGACGCACGTCGCGACCAGTGGTGGTTGATTGCTCAACTCGCTTGCTGTCCGTCGTGCCCCGATGCGTCGTCCGACCGGGAATCACGA

>T5558

CCCCGCTCGAACGGAGCGGGTGCGGCGGGGGCGGATGTTGGCCTCCCGTGCGTCCCCGTGCGGTTGGCCCAAATTGGATCCCCCGGCGACGCACGTCGCGACCAGTGGTGGTTGATTGCTCAACTCGCTTGCTGTCCGTCGTGCCCCGATGCGTCGTCCGACCGGGAATCACGA

>T5559

TTGCAGAATCCCGTGAACCATCGAGTCTTTGAACGCAAGTTGCGCCCGAAGCCTTCGGGCCGAGGGCACGCCTGCCTGGGCGTCACGCATCGCGTCGCCCCCCCTACCCCGCTCGAACGGAGCGGGTGCGGCGGGGGCGGATGTTGGCCTCCCGTGCGTCCCCGTGCGGTTGGCCCAAATTGGATCCCCCGGCGACGCACGTCGCGACCAGTGGTGGTTGATTGCTCAACTCGCTTGCTGTCCGTCGTGCCCCGATGCGTCGTCCGACCGGGAATCACGA

>T5560

CCCCGCTCGAACGGAGCGGGTGCGGCGGGGGCGGATGTTGGCCTCCCGTGCGTCCCCGTGCGGTTGGCCCAAATTGGATCCCCCGGCGACGCACGTCGCGACCAGTGGTGGTTGATTGCTCAACTCGCTTGCTGTCCGTCGTGCCCCGATGCGTCGTCCGACCGGGAATCACGA

>T5562

TGCGATACTTGGTGTGAATTGCAGAATCCCGTGAACCATCGAGTCTTTGAACGCAAGTTGCGCCCCAAGCCGTTAGGTCGAGGGCACGTCTGCCTGGGTGTCACGCATCGTTGCCCCACCCCATCCCCTCGGGGCCATGGTGGTGTGGGCGGAAAATGGCCTCCCGTGGGCTATTCGCTTGCGGTTGGCCGAAAAATGAGTCATCGGCGACCGAAGCCGCGACGATCGGTGGTGAAAACAAGCCTCTCGAGCTCCCGTCGCGTGCCCGTGTCGACGAAATAGTGCTCAAGGACCCTGACGCTCCGCGTAAGCGGCGCTCGCATCGCGACCCCAGGTCAGGCGG

>T5563

GGCGGGGGCGGATGTTGGCCTCCCGTGCGTCCCCGTGCGGTTGGCCCAAATTGGATCCCCCGGCGACGCACGTCGCGACCAGTGGTGGTTGATTGCTCAACTCGCTTGCTGTCCGTCGTGCCCCGATGCGTCGTCCGACCGGGAATCACGA

>T5565

TTGCAGAATCCCGTGAACCATCGAGTCTTTGAACGCAAGTTGCGCCCGAAGCCTTCGGGCCGAGGGCACGCCTGCCTGGGCGTCACGCATCGCGTCGCCCCCCCTACCCCGCTCGAACGGAGCGGGTGCGGCGGGGGCGGATGTTGGCCTCCCGTGCGTCCCCGTGCGGTTGGCCCAAATTGGATCCCCCGGCGACGCACGTCGCGACCAGTGGTGGTTGATTGCTCAACTCGCTTGCTGTCCGTCGTGCCCCGATGCGTCGTCCGACCGGGAATCACGAGAGACCCAAGGCGCCGAGCGCGCTTCCGACAGCGACCCCAG

>T5566

CCCCGCTCGAACGGAGCGGGTGCGGCGGGGGCGGATGTTGGCCTCCCGTGCGTCCCCGTGCGGTTGGCCCAAATTGGATCCCCCGGCGACGCACGTCGCGACCAGTGGTGGTTGATTGCTCAACTCGCTTGCTGTCCGTCGTGCCCCGATGCGTCGTCCGACCGGGAATCACGA

>T5566

CCCCGCTCGAACGGAGCGGGTGCGGCGGGGGCGGATGTTGGCCTCCCGTGCGTCCCCGTGCGGTTGGCCCAAATTGGATCCCCCGGCGACGCACGTCGCGACCAGTGGTGGTTGATTGCTCAACTCGCTTGCTGTCCGTCGTGCCCCGATGCGTCGTCCGACCGGGAATCACGA

>T5567

TGCGATACTTGGTGTGAATTGCAGAATCCCGTGAACCATCGAGTCTTTGAACGCAAGTTGCGCCCGAAGCCTTCGGGCCGAGGGCACGCCTGCCTGGGCGTCACGCATCGCGTCGCCCCCCCTACCCCGCTCGAACGGAGCGGGTGCGGCGGGGGCGGATGTTGGCCTCCCGTGCGTCCCCGTGCGGTTGGCCCAAATTGGATCCCCCGGCGACGCACGTCGCGACCAGTGGTGGTTGATTGCTCAACTCGCTTGCTGTCCGTCGTGCCCCGATGCGTCGTCCGACCGGGAATCACGA

>T5567

TGCGATACTTGGTGTGAATTGCAGAATCCCGTGAACCATCGAGTCTTTGAACGCAAGTTGCGCCCGAAGCCTTCGGGCCGAGGGCACGCCTGCCTGGGCGTCACGCATCGCGTCGCCCCCCCTCCCCCGCTCGAACGGAGCGGGTGCGGCGGGGGCGGATGTTGGCCTCCCGTGCGTCCCCGTGCGGTTGGCCCAAATTGGATCCCCCGGCGACGCACGTCGCGACCAGTGGTGGTTGATTGCTCAACTCGCTTGCTGTCCGTCGTGCCCCGATGCGTCGTCCGACCGGGAATCACGA

>T5568

TGCGATACTTGGTGTGAATTGCAGAATCCCGTGAACCATCGAGTCTTTGAACGCAAGTTGCGCCCGAAGCCTTCGGGCCGAGGGCACGCCTGCCTGGGCGTCACGCATCGCGTCGCCCCCCCTACCCCGCTCGAACGGAGCGGGTGCGGCGGGGGCGGATGTTGGCCTCCCGTGCGTCCCCGTGCGGTTGGCCCAAATTGGATCCCCCGGCGACGCACGTCGCGACCAGTGGTGGTTGATTGCTCAACTCGCTTGCTGTCCGTCGTGCCCCGATGCGTCGTCCGACCGGGAATCACGA

>T5568

TGCGATACTTGGTGTGAATTGCAGAATCCCGTGAACCATCGAGTCTTTGAACGCAAGTTGCGCCCGAAGCCTTCGGGCCGAGGGCACGCCTGCCTGGGCGTCACGCATCGCGTCGCCCCCCCTCCCCCGCTCGAACGGAGCGGGTGCGGCGGGGGCGGATGTTGGCCTCCCGTGCGTCCCCGTGCGGTTGGCCCAAATTGGATCCCCCGGCGACGCACGTCGCGACCAGTGGTGGTTGATTGCTCAACTCGCTTGCTGTCCGTCGTGCCCCGATGCGTCGTCCGACCGGGA

>T5569

TGCGATACTTGGTGTGAATTGCAGAATCCCGTGAACCATCGAGTCTTTGAACGCAAGTTGCGCCCTAAGCCTTCTGGCCGAGGGCACGTCTGCCTGGGTGTCACAAATCGTCGTCCCCCCATCCTCTCGAGGATAATGGACGGAAGCTGGTCTCCCGTGTGTTACCGCACGCGGTTGGCCAAAATCCGAGCTAAGGACGCAAGGAGCGTCTCGACATGCGGTGGTGAATTAAAACCTCGTCATACCGTTGGCCGCTCCTGTCCTGATGCTCTCGATGACCCAAAGTCCTCAACGCGACCCCAG

>T5570

CCCCCCCTCCCCCGCTCGAACGGAGCGGGTGCGGCGGGGGCGGATGTTGGCCTCCCGTGCGTCCCCGTGCGGTTGGCCCAAATTGGATCCCCCGGCGAC

>T5571

TGCGATACTTGGTGTGAATTGCAGAATCCCGTGAACCATCGAGTCTTTGAACGCAAGTTGCGCCCGAAGCCTTCGGGCCGAGGGCACGCCTGCCTGGGCGTCACGCATCGCGTCGCCCCCCCTACCCCGCTCGAACGGAGCGGGTGCGGCGGGGGCGGATGTTGGCCTCCCGTGCGTCCCCGTGCGGTTGGCCCAAATTGGATCCCCCGGCGACGCACGTCGCGACCAGTGGTGGTTGATTGCTCAACTCGCTTGCTGTCCGTCGTGCCCCGATGCGTCGTCCGACCGGGAATCACGA

>T5572

TGCGATACTTGGTGTGAATTGCAGAATCCCGTGAACCATCGAGTCTTTGAACGCAAGTTGCGCCCGAAGCCTTCGGGCCGAGGGCACGCCTGCCTGGGCGTCACGCATCGCGTCGCCCCCCCTCCCCCGCTCGAACGGAGCGGGTGCGGCGGGGGCGGATGTTGGCCTCCCGTGCGTCCCCGTGCGGTTGGCCCAAATTGGATCCCCCGGCGACGCACGTCGCGACCAGTGGTGGTTGATTGCTCAACTCGCTTGCTGTCCGTCGTGCCCCGATGCGTCGTCCGACCGGGAATCACGA

**3b. DNA sequences of Banlangen samples at the *rbcL* region (primers rbcLaF/rbcLaR)**

>T5557

GAGTACAAATTGACTTATTATACTCCTGAATATGAAACCAAAGATACTGATATCTTGGCAGCATTCCGAGTAACTCCTCAACCGGGAGTTCCACCTGAAGAAGCAGGGGCCGCGGTAGCTGCCGAATCCTCCACCGGTACATGGACAACCGTGTGGACCGATGGACTTACCAGCCTTGATCGTTACAAAGGGCGATGCTACAACATCGAGGCCGTTCCTGGCGAAGCAGATCAATACATCTGTTATGTAGCTTACCCTTTAGACCTTTTTGAAGAAGGTTCTGTTACCAACATGTTTACTTCCATTGTAGGAAATGTTTTTGGATTCAAAGCACTGCGTGCTCTACGTCTGGAAGATCTGCGAATCCCTGTTGCTTATGTTAAAACTTTCCAGGGCCCGCCTCATGGGATCCAAAGTGAGAGAGATAAATTGAACAAGTATGGTCGTCCTCTGCTGGGATGTACTATTAAACCTAAATTGGGGTTATCCGCTAAAA

>T5560

GAGTACAAATTGACTTATTATACTCCTGAATATGAAACCAAAGATACTGATATCTTGGCAGCATTCCGAGTAACTCCTCAACCGGGAGTTCCACCTGAAGAAGCAGGGGCCGCGGTAGCTGCCGAATCCTCCACCGGTACATGGACAACCGTGTGGACCGATGGACTTACCAGCCTTGATCGTTACAAAGGGCGATGCTACAACATCGAGCCCGTTCCTGGCGAAGCAGATCAATACATCTGTTATGTAGCTTACCCTTTAGACCTTTTTGAAGAAGGTTCTGTTACCAACATGTTTACTTCCATTGTAGGAAATGTTTTTGGATTCAAAGCACTGCGTGCTCTACGTCTGGAAGATCTGCGAATCCCTGTTGCTTATGTTAAAACTTTCCAGGGCCCGCCTCATGGGATCCAAAGTGAGAGAGATAAATTGAACAAGTATGGTCGTCCTCTGCTGGGATGTACTATTAAACCTAAATTGGGGTTATCCGCTAAAA

>T5561

GAGTACAAATTGACTTATTATACTCCTGAATATGAAACCAAAGATACTGATATCTTGGCAGCATTCCGAGTAACTCCTCAACCGGGAGTTCCACCTGAAGAAGCAGGGGCCGCGGTAGCTGCCGAATCCTCCACCGGTACATGGACAACCGTGTGGACCGATGGACTTACCAGCCTTGATCGTTACAAAGGGCGATGCTACAACATCGAGGCCGTTCCTGGCGAAGCAGATCAATACATCTGTTATGTAGCTTACCCTTTAGACCTTTTTGAAGAAGGTTCTGTTACCAACATGTTTACTTCCATTGTAGGAAATGTTTTTGGATTCAAAGCACTGCGTGCTCTACGTCTGGAAGATCTGCGAATCCCTGTTGCTTATGTTAAAACTTTCCAGGGCCCGCCTCATGGGATCCAAAGTGAGAGAGATAAATTGAACAAGTATGGTCGTCCTCTGCTGGGATGTACTATTAAACCTAAATTGGGGTTATCCGCTAAAA

>T5562

AAATTGACTTATTATACTCCTGAATATGAAACCAAAGATACTGATATCTTGGCAGCATTCCGAGTAACTCCTCAACCGGGAGTTCCACCTGAAGAAGCAGGGGCCGCGGTAGCTGCCGAATCCTCCACCGGTACATGGACAACCGTGTGGACCGATGGACTTACCAGCCTTGATCGTT

>T5563

GAGTACAAATTGACTTATTATACTCCTGAATATGAAACCAAAGATACTGATATCTTGGCAGCATTCCGAGTAACTCCTCAACCGGGAGTTCCACCTGAAGAAGCAGGGGCCGCGGTAGCTGCCGAATCCTCCACCGGTACATGGACAACCGTGTGGACCGATGGACTTACCAGCCTTGATCGTTACAAAGGGCGATGCTACAACATCGAGCCCGTTCCTGGCGAAGCAGATCAATACATCTGTTATGTAGCTTACCCTTTAGACCTTTTTGAAGAAGGTTCTGTTACCAACATGTTTACTTCCATTGTAGGAAATGTTTTTGGATTCAAAGCACTGCGTGCTCTACGTCTGGAAGATCTGCGAATCCCTGTTGCTTATGTTAAAACTTTCCAGGGCCCGCCTCATGGGATCCAAAGTGAGAGAGATAAATTGAACAAGTATGGTCGTCCTCTGCTGGGATGTACTATTAAACCTAAATTGGGGTTATCCGCTAAAA

>T5564

GAGTACAAATTGACTTATTATACTCCTGAATATGAAACCAAAGATACTGATATCTTGGCAGCATTCCGAGTAACTCCTCAACCGGGAGTTCCACCTGAAGAAGCAGGGGCCGCGGTAGCTGCCGAATCCTCCACCGGTACATGGACAACCGTGTGGACCGATGGACTTACCAGCCTTGATCGTTACAAAGGGCGATGCTACAACATCGAGCCCGTTCCTGGCGAAGCAGATCAATACATCTGTTATGTAGCTTACCCTTTAGACCTTTTTGAAGAAGGTTCTGTTACCAACATGTTTACTTCCATTGTAGGAAATGTTTTTGGATTCAAAGCACTGCGTGCTCTACGTCTGGAAGATCTGCGAATCCCTGTTGCTTATGTTAAAACTTTCCAGGGCCCGCCTCATGGGATCCAAAGTGAGAGAGATAAATTGAACAAGTATGGTCGTCCTCTGCTGGGATGTACTATTAAACCTAAATTGGGGTTATCCGCTAAAA

>T5564

GAGTACAAATTGACTTATTATACTCCTGAATATGAAACCAAAGATACTGATATCTTGGCAGCATTCCGAGTAACTCCTCAACCGGGAGTTCCACCTGAAGAAGCAGGGGCCGCGGTAGCTGCCGAATCCTCCACCGGTACATGGACAACCGTGTGGACCGATGGACTTACCAGCCTTGATCGTTACAAAGGGCGATGCTACAACATCGAGCCCGTTCCTGGCGAAGCAGATCAATACATCTGTTATGTAGCTTACCCTTTAGACCTTTTTGAAGAAGGTTCTGTTACCAACATGTTTACTTCCATTGTAGGAAATGTTTTTGGATTCAAAGCACTGCGTGCTCTACGTCTGGAAGATCTGCGAATCCCTGTTGCTTATGTTAAAACTTTCCAGGGCCCGCCTCATGGGATCCAAAGTGAGAGAGATAAATTGAACAAGTATGGTCGTCCTCTGCTGGGATGTACTATTAAACCTAAATTGGGGTTATCCGCTAAAAACTATGGTAGAGCGT

>T5566

GAGTACAAATTGACTTATTATACTCCTGAATATGAAACCAAAGATACTGATATCTTGGCAGCATTCCGAGTAACTCCTCAACCGGGAGTTCCACCTGAAGAAGCAGGGGCCGCGGTAGCTGCCGAATCCTCCACCGGTACATGGACAACCGTGTGGACCGATGGACTTACCAGCCTTGATCGTTACAAAGGGCGATGCTACAACATCGAGCCCGTTCCTGGCGAAGCAGATCAATACATCTGTTATGTAGCTTACCCTTTAGACCTTTTTGAAGAAGGTTCTGTTACCAACATGTTTACTTCCATTGTAGGAAATGTTTTTGGATTCAAAGCACTGCGTGCTCTACGTCTGGAAGATCTGCGAATCCCTGTTGCTTATGTTAAAACTTTCCAGGGCCCGCCTCATGGGATCCAAAGTGAGAGAGATAAATTGAACAAGTATGGTCGTCCTCTGCTGGGATGTACTATTAAACCTAAATTGGGGTTATCCGCTAAAA

>T5567

GAGTACAAATTGACTTATTATACTCCTGAATATGAAACCAAAGATACTGATATCTTGGCAGCATTCCGAGTAACTCCTCAACCGGGAGTTCCACCTGAAGAAGCAGGGGCCGCGGTAGCTGCCGAATCCTCCACCGGTACATGGACAACCGTGTGGACCGATGGACTTACCAGCCTTGATCGTTACAAAGGGCGATGCTACAACATCGAGCCCGTTCCTGGCGAAGCAGATCAATACATCTGTTATGTAGCTTACCCTTTAGACCTTTTTGAAGAAGGTTCTGTTACCAACATGTTTACTTCCATTGTAGGAAATGTTTTTGGATTCAAAGCACTGCGTGCTCTACGTCTGGAAGATCTGCGAATCCCTGTTGCTTATGTTAAAACTTTCCAGGGCCCGCCTCATGGGATCCAAAGTGAGAGAGATAAATTGAACAAGTATGGTCGTCCTCTGCTGGGATGTACTATTAAACCTAAATTGGGGTTATCCGCTAAAAACTATG

>T5568

GAGTACAAATTGACTTATTATACTCCTGAATATGAAACCAAAGATACTGATATCTTGGCAGCATTCCGAGTAACTCCTCAACCGGGAGTTCCACCTGAAGAAGCAGGGGCCGCGGTAGCTGCCGAATCCTCCACCGGTACATGGACAACCGTGTGGACCGATGGACTTACCAGCCTTGATCGTTACAAAGGGCGATGCTACAACATCGAGCCCGTTCCTGGCGAAGCAGATCAATACATCTGTTATGTAGCTTACCCTTTAGACCTTTTTGAAGAAGGTTCTGTTACCAACATGTTTACTTCCATTGTAGGAAATGTTTTTGGATTCAAAGCACTGCGTGCTCTACGTCTGGAAGATCTGCGAATCCCTGTTGCTTATGTTAAAACTTTCCAGGGCCCGCCTCATGGGATCCAAAGTGAGAGAGATAAATTGAACAAGTATGGTCGTCCTCTGCTGGGATGTACTATTAAACCTAAATTGGGGTTATCCGCTAAAA

>T5570

GAGTACAAATTGACTTATTATACTCCTGAATATGAAACCAAAGATACTGATATCTTGGCAGCATTCCGAGTAACTCCTCAACCGGGAGTTCCACCTGAAGAAGCAGGGGCCGCGGTAGCTGCCGAATCCTCCACCGGTACATGGACAACCGTGTGGACCGATGGACTTACCAGCCTTGATCGTTACAAAGGGCGATGCTACAACATCGAGCCCGTTCCTGGCGAAGCAGATCAATACATCTGTTATGTAGCTTACCCTTTAGACCTTTTTGAAGAAGGTTCTGTTACCAACATGTTTACTTCCATTGTAGGAAATGTTTTTGGATTCAAAGCACTGCGTGCTCTACGTCTGGAAGATCTGCGAATCCCTGTTGCTTATGTTAAAACTTTCCAGGGCCCGCCTCATGGGATCCAAAGTGAGAGAGATAAATTGAACAAGTATGGTCGTCCTCTGCTGGGATGTACTATTAAACCTAAATTGGGGTTATCCGCTAAAA

>T5572

GAGTACAAATTGACTTATTATACTCCTGAATATGAAACCAAAGATACTGATATCTTGGCAGCATTCCGAGTAACTCCTCAACCGGGAGTTCCACCTGAAGAAGCAGGGGCCGCGGTAGCTGCCGAATCCTCCACCGGTACATGGACAACCGTGTGGACCGATGGACTTACCAGCCTTGATCGTTACAAAGGGCGATGCTACAACATCGAGCCCGTTCCTGGCGAAGCAGATCAATACATCTGTTATGTAGCTTACCCTTTAGACCTTTTTGAAGAAGGTTCTGTTACCAACATGTTTACTTCCATTGTAGGAAATGTTTTTGGATTCAAAGCACTGCGTGCTCTACGTCTGGAAGATCTGCGAATCCCTGTTGCTTATGTTAAAACTTTCCAGGGCCCGCCTCATGGGATCCAAAGTGAGAGAGATAAATTGAACAAGTATGGTCGTCCTCTGCTGGGATGTACTATTAAACCTAAATTGGGGTTATCCGCTAAAA

>T5573

GAGTACAAATTGACTTATTATACTCCTGAATATGAAACCAAAGATACTGATATCTTGGCAGCATTCCGAGTAACTCCTCAACCGGGAGTTCCACCTGAAGAAGCAGGGGCCGCGGTAGCTGCCGAATCCTCCACCGGTACATGGACAACCGTGTGGACCGATGGACTTACCAGCCTTGATCGTTACAAAGGGCGATGCTACAACATCGAGGCCGTTCCTGGCGAAGCAGATCAATACATCTGTTATGTAGCTTACCCTTTAGACCTTTTTGAAGAAGGTTCTGTTACCAACATGTTTACTTCCATTGTAGGAAATGTTTTTGGATTCAAAGCACTGCGTGCTCTACGTCTGGAAGATCTGCGAATCCCTGTTGCTTATGTTAAAACTTTCCAGGGCCCGCCTCATGGGATCCAAAGTGAGAGAGATAAATTGAACAAGTATGGTCGTCCTCTGCTGGGATGTACTATTAAACCTAAATTGGGGTTATCCGCTAAAA

>T5574

GAGTACAAATTGACTTATTATACTCCTGAATATGAAACCAAAGATACTGATATCTTGGCAGCATTCCGAGTAACTCCTCAACCGGGAGTTCCACCTGAAGAAGCAGGGGCCGCGGTAGCTGCCGAATCCTCCACCGGTACATGGACAACCGTGTGGACCGATGGACTTACCAGCCTTGATCGTTACAAAGGGCGATGCTACAACATCGAGCCCGTTCCTGGCGAAGCAGATCAATACATCTGTTATGTAGCTTACCCTTTAGACCTTTTTGAAGAAGGTTCTGTTACCAACATGTTTACTTCCATTGTAGGAAATGTTTTTGGATTCAAAGCACTGCGTGCTCTACGTCTGGAAGATCTGCGAATCCCTGTTGCTTATGTTAAAACTTTCCAGGGCCCGCCTCATGGGATCCAAAGTGAGAGAGATAAATTGAACAAGTATGGTCGTCCTCTGCTGGGATGTACTATTAAACCTAAATTGGGGTTATCCGCTAAAAACTATG

>T5574

GAGTACAAATTGACTTATTATACTCCTGAATATGAAACCAAAGATACTGATATCTTGGCAGCATTCCGAGTAACTCCTCAACCGGGAGTTCCACCTGAAGAAGCAGGGGCCGCGGTAGCTGCCGAATCCTCCACCGGTACATGGACAACCGTGTGGACCGATGGACTTACCAGCCTTGATCGTTACAAAGGGCGATGCTACAACATCGAGCCCGTTCCTGGCGAAGCAGATCAATACATCTGTTATGTAGCTTACCCTTTAGACCTTTTTGAAGAAGGTTCTGTTACCAACATGTTTACTTCCATTGTAGGAAATGTTTTTGGATTCAAAGCACTGCGTGCTCTACGTCTGGAAGATCTGCGAATCCCTGTTGCTTATGTTAAAACTTTCCAGGGCCCGCCTCATGGGATCCAAAGTGAGAGAGATAAATTGAACAAGTATGGTCGTCCTCTGCTGGGATGTACTATTAAACCTAAATTGGGGTTATCCGCTA

>T5624

GAGTACAAATTGACTTATTATACTCCTGAATATGAAACCAAAGATACTGATATCTTGGCAGCATTCCGAGTAACTCCTCAACCGGGAGTTCCACCTGAAGAAGCAGGGGCCGCGGTAGCTGCCGAATCCTCCACCGGTACATGGACAACCGTGTGGACCGATGGACTTACCAGCCTTGATCGTTACAAAGGGCGATGCTACAACATCGAGCCCGTTCCTGGCGAAGCAGATCAATACATCTGTTATGTAGCTTACCCTTTAGACCTTTTTGAAGAAGGTTCTGTTACCAACATGTTTACTTCCATTGTAGGAAATGTTTTTGGATTCAAAGCACTGCGTGCTCTACGTCTGGAAGATCTGCGAATCCCTGTTGCTTATGTTAAAACTTTCCAGGGCCCGCCTCATGGGATCCAAAGTGAGAGAGATAAATTGAACAAGTATGGTCGTCCTCTGCTGGGATGTACTATTAAACCTAAATTGGGGTTATCCGCTAAAAACTATGGTAGAGCGT
