# Supplementary material for: Quality assessment of medicinal material Daqingye and Banlangen from Isatis tinctoria Fort. reveals widespread substitution with Strobilanthes species
Source: PLoS One. 2025 May 7;20(5):e0323084. doi: 10.1371/journal.pone.0323084 (PMC12058189; doi:10.1371/journal.pone.0323084)
Supplement: S4 File — (DOCX) [file pone.0323084.s004.docx]

**S4 File. DNA sequences of Daqingye samples**

**4a. DNA sequences of samples at the *ITS2* region (primers ITS2F/ITS3R)**

>T5537

CGCAAGTTGCGCCCGAAGCCATTCGGTCGAGGGCACATCTGCCTGGGCGTCACGTCTCGCGTCGCTCCTAAACCATGTCTCCTCAAAAAGGATGTGCGAGATAGGGGCGGATACTGGTCTCCCGTGCCTATGGTGTGGTTGGCCGAAATTACGAGTCTCCTTTTCATGGACACACGGCAAGTGGTGGTTGAGCTGACCTTAGTTTCGTGTTTGTGTGTTCGTAGATGTATTGGAAGACCTAGCAAAAGTACCCTAGTGCGTCGTCTTGCGGCGGTGCTTCGTCCGCGACCCCAGGTCAGGTGGGACTACCCGCTGAGTTTAAGCATATCAATAAGCGGAGGAGAAGAAACTTACAAGGATTCCCTTAGTAACGGCGAGCGAACCGGGAATAGCCCAGCT

>T5543

ACGCAAGTTGCGCCCTAAGCCTTCTGGCCGAGGGCACGTCTGCCTGGGTGTCACAAATCGTCGTCCCCCCATCCTCTCGAGGATAATGGACGGAAGCTGGTCTCCCGTGTGTTACCGCACGCGGTTGGCCAAAATCCGAGCTAAGGACGCAAGGAGCGTCTCGACATGCGGTGGTGAATTAAAACCTCGTCATACCGTTGGCCGCTCCTGTCCTGATGCTCTCGATGACCCAAAGTCCTCAACGCGACCCCAGGTCAGGCGGGATCACCCGCTGAGTTTAAGCATATCAATAAGCGGAGGAAAAGAAACTAACAAGGATTCCCTTAGTAACGGCGAGCGAACCGGGAAGAGCCCAGCT

>T5543

CGCAAGTTGCGCCCTAAGCCTTCTGGCCGAGGGCACGTCTGCCTGGGTGTCACAAATCGTCGTCCCCCCATCCTCTCGAGGATAATGGACGGAAGCTGGTCTCCCGTGTGTTACCGCACGCGGTTGGCCAAAATCCGAGCTAAGGACGCAAGGAGCGTCTCGACATGCGGTGGTGAATTAAAACCTCGTCATACCGTTGGCCGCTCCTGTCCTGATGCTCTCGATGACCCAAAGTCCTCAACGCGACCCCAGGTCAGGCGGGATCACCCGCTGAGTTTAAGCATATCAATAAGCGGAGGAAAAGAAACTAACAAGGATTCCCTTAGTAACGGCGAGCGAACCGGGAAGAGCCCAGCT

**4b. DNA sequences at the *matK* region (primers KIM3F/KIM1R)**

>T5529

GAAAGTCGAAGTATATATTTTATTTGATACAAACTCTTTTTTATAGAAGATCCGCTATGATAATGAGAAAGATTTCTGCATATACGCCCAAATAGGGCGATAATATTAGAATCTGATAAATCAGCCCAAACCGGCTTACTAATAGGGTGCCCTAATACGTTACAAAATTTTTCTTTAGCCAATGCCGCAATCAGAGGAATAATTGGAATAAAAATATCGAACTTCTTAATGGCATTATTAATTGGAAATGCATTTTCGAGGATTTGGCTCCGTACCACTGAAGGTTTCATCAGCACACGTGAAAGATAGCCAAGAAATTCGAGGAAATGATTGGATAATTGCTTTCTAGAAATCCTTCTTGATTGAAACCACAGCGAAAAATGCCATTGCCAAAAAGCGACAAGGTAACATTTCCATTTATTCATAAAATGGAACGTCCCTTTTGAAGCCAAGAGGAATCTTCTTTGATACCTAATATAATGCATGCAAGGTTTCTTGACCAACCATAGGTTCACCTGAAAATCCTTAACCTTAACAAAGACGTTCACAAGATGTTCTATTTTTCCATGGAAATCGATTCGTTCAAGAAAACCTCCAGAAGATGTTGATCGTAAATGAGAAGATTGGTTACGGAAAAAGAAGAAAGTGGATTCATATTCCCATACATGAGAATTATCTAAGAATAAGAATAACCTTAGATTTCTTTTTGAAAAAGAGTAATTGGCTTTCTTTGGCCTAATTAAAAGATTCCAATTGCAATACTCGTTCATAAAAAATCGTAAAAAATGCAAAGAAGAGGCATCTTT

>T5532

AAGTCGAAGTATATATTTTATTTGATACAAACTCTTTTTTATAGAAGATCCGCTATGATAATGAGAAAGATTTCTGCATATACGCCCAAATAGGGCGATAATATTAGAATCTGATAAATCAGCCCAAACCGGCTTACTAATAGGGTGCCCTAATACGTTACAAAATTTTTCTTTAGCCAATGCCGCAATCAGAGGAATAATTGGAATAAAAATATCGAACTTCTTAATGGCATTATTAATTGGAAATGCATTTTCGAGGATTTGGCTCCGTACCACTGAAGGTTTCATCAGCACACGTGAAAGATAGCCAAGAAATTCGAGGAAATGATTGGATAATTGCTTTCTAGAAATCCTTCTTGATTGAAACCACAGCGAAAAATGCCATTGCCAAAAAGCGACAAGGTAACATTTCCATTTATTCATAAAATGGAACGTCCCTTTTGAAGCCAAGAGGAATCTTCTTTGATACCTAATATAATGCATGCAAGGTTTCTTGACCAACCATAGGTTCACCTGAAAATCCTTAACCTTAACAAAGACGTTCACAAGATGTTCTATTTTTCCATGGAAATCGATTCGTTCAAGAAAACCTCCAGAAGATGTTGATCGTAAATGAGAAGATTGGTTACGGAAAAAGAAGAAAGTGGATTCATATTCCCATACATGAGAATTATCTAAGAATAAGAATAACCTTAGATTTCTTTTTGAAAAAGAGTAATTGGCTTTCTTTGGCCTAATTAAAAGATTCCAATTGCAATACTCGTTCATAAAAAATCGTAAAAA

>T5532

GTATATATTTTATTTGATACAAACTCTTTTTTATAGAAGATCCGCTATGATAATGAGAAAGATTTCTGCATATACGCCCAAATAGGGCGATAATATTAGAATCTGATAAATCAGCCCAAACCGGCTTACTAATAGGGTGCCCTAATACGTTACAAAATTTTTCTTTAGCCAATGCCGCAATCAGAGGAATAATTGGAATAAAAATATCGAACTTCTTAATGGCATTATTAATTGGAAATGCATTTTCGAGGATTTGGCTCCGTACCACTGAAGGTTTCATCAGCACACGTGAAAGATAGCCAAGAAATTCGAGGAAATGATTGGATAATTGCTTTCTAGAAATCCTTCTTGATTGAAACCACAGCGAAAAATGCCATTGCCAAAAAGCGACAAGGTAACATTTCCATTTATTCATAAAATGGAACGTCCCTTTTGAAGCCAAGAGGAATCTTCTTTGATACCTAATATAATGCATGCAAGGTTTCTTGACCAACCATAGGTTCACCTGAAAATCCTTAACCTTAACAAAGACGTTCACAAGATGTTCTATTTTTCCATGGAAATCGATTCGTTCAAGAAAACCTCCAGAAGATGTTGATCGTAAATGAGAAGATTGGTTACGGAAAAAGAAGAAAGTGGATTCATATTCCCATACATGAGAATTATCTAAGAATAAGAATAACCTTAGATTTCTTTTTGAAAAAGAGTAATTGGCTTTCTTTGGCCTAATTAAAAGATTCCAATTGCAATACTCGTTCATAAAAAATCGTAAAAAATGCAAAGAAGAGGCATCT

>T5536

GAGAAAGATTTCTGCATATACGCCCAAATAGGGCGATAATATTAGAATCTGATAAATCAGCCCAAACCGGCTTACTAATAGGGTGCCCTAATACGTTACAAAATTTTTCTTTAGCCAATGCCGCAATCAGAGGAATAATTGGAATAAAAATATCGAACTTCTTAATGGCATTATTAATTGGAAATGCATTTTCGAGGATTTGGCTCCGTACCACTGAAGGTTTCATCAGCACACGTGAAAGATAGCCAAGAAATTCGAGGAAATGATTGGATAATTGCTTTCTAGAAATCCTTCTTGATTGAAACCACAGCGAAAAATGCCATTGCCAAAAAGCGACAAGGTAACATTTCCATTTATTCATAAAATGGAACGTCCCTTTTGAAGCCAAGAGGAATCTTCTTTGATACCTAATATAATGCATGCAAGGTTTCTTGACCAACCATAGGTTCACCTGAAAATCCTTAACCTTAACAAAGACGTTCACAAGATGTTCTATTTTTCCATGGAAATCGATTCGTTCAAGAAAACCTCCAGAAGATGTTGATCGTAAATGAGAAGATTGGTTACGGAAAAAGAAGAAAGTGGATTCATATTCCCATACATGAGAATTATCTAAGAATAAGAATAACCTTAGATTTCTTTTTGAAAAAGAGTAATTGGCTTTCTTTGGCCTAATTAAAAGATTCCAATTGCAATACTCGTTCATAAAAAATCGTAAAAAATGCAAAGAAGAGGCATCTTT

>T5536

GTATATATTTTATTTGATACAAACTCTTTTTTATAGAAGATCCGCTATGATAATGAGAAAGATTTCTGCATATACGCCCAAATAGGGCGATAATATTAGAATCTGATAAATCAGCCCAAACCGGCTTACTAATAGGGTGCCCTAATACGTTACAAAATTTTTCTTTAGCCAATGCCGCAATCAGAGGAATAATTGGAATAAAAATATCGAACTTCTTAATGGCATTATTAATTGGAAATGCATTTTCGAGGATTTGGCTCCGTACCACTGAAGGTTTCATCAGCACACGTGAAAGATAGCCAAGAAATTCGAGGAAATGATTGGATAATTGCTTTCTAGAAATCCTTCTTGATTGAAACCACAGCGAAAAATGCCATTGCCAAAAAGCGACAAGGTAACATTTCCATTTATTCATAAAATGGAACGTCCCTTTTGAAGCCAAGAGGAATCTTCTTTGATACCTAATATAATGCATGCAAGGTTTCTTGACCAACCATAGGTTCACCTGAAAATCCTTAACCTTAACAAAGACGTTCACAAGATGTTCTATTTTTCCATGGAAATCGATTCGTTCAAGAAAACCTCCAGAAGATGTTGATCGTAAATGAGAAGATTGGTTACGGAAAAAGAAGAAAGTGGATTCATATTCCCATACATGAGAATTATCTAAGAATAAGAATAACCTTAGATTTCTTTTTGAAAAAGAGTAATTGGCTTTCTTTGGCCTAATTAAAAGATTCCAATTGCAATACTCGTTCATAAAAAATCGTAAAAAATGCAAAGAAGAGGCATCTTT

>T5538

AAGTCGAAGTATATATTTTATTTGATACAAACTCTTTTTTATAGAAGATCCGCTATGATAATGAGAAAGATTTCTGCATATACGCCCAAATAGGGCGATAATATTAGAATCTGATAAATCAGCCCAAACCGGCTTACTAATAGGGTGCCCTAATACGTTACAAAATTTTTCTTTAGCCAATGCCGCAATCAGAGGAATAATTGGAATAAAAATATCGAACTTCTTAATGGCATTATTAATTGGAAATGCATTTTCGAGGATTTGGCTCCGTACCACTGAAGGTTTCATCAGCACACGTGAAAGATAGCCAAGAAATTCGAGGAAATGATTGGATAATTGCTTTCTAGAAATCCTTCTTGATTGAAACCACAGCGAAAAATGCCATTGCCAAAAAGCGACAAGGTAACATTTCCATTTATTCATAAAATGGAACGTCCCTTTTGAAGCCAAGAGGAATCTTCTTTGATACCTAATATAATGCATGCAAGGTTTCTTGACCAACCATAGGTTCACCTGAAAATCCTTAACCTTAACAAAGACGTTCACAAGATGTTCTATTTTTCCATGGAAA

>T5540

GTATATATTTTATTTGATACAAACTCTTTTTTATAGAAGATCCGCTATGATAATGAGAAAGATTTCTGCATATACGCCCAAATAGGGCGATAATATTAGAATCTGATAAATCAGCCCAAACCGGCTTACTAATAGGGTGCCCTAATACGTTACAAAATTTTTCTTTAGCCAATGCCGCAATCAGAGGAATAATTGGAATAAAAATATCGAACTTCTTAATGGCATTATTAATTGGAAATGCATTTTCGAGGATTTGGCTCCGTACCACTGAAGGTTTCATCAGCACACGTGAAAGATAGCCAAGAAATTCGAGGAAATGATTGGATAATTGCTTTCTAGAAATCCTTCTTGATTGAAACCACAGCGAAAAATGCCATTGCCAAAAAGCGACAAGGTAACATTTCCATTTATTCATAAAATGGAACGTCCCTTTTGAAGCCAAGAGGAATCTTCTTTGATACCTAATATAATGCATGCAAGGTTTCTTGACCAACCATAGGTTCACCTGAAAATCCTTAACCTTAACAAAGACGTTCACAAGATGTTCTATTTTTCCATGGAAATCGATTCGTTCAAGAAAACCTCCAGAAGATGTTGATCGTAAATGAGAAGATTGGTTACGGAAAAAGAAGAAAGTGGATTCATATTCCCATACATGAGAATTATCTAAGAATAAGAATAACCTTAGATTTCTTTTTGAAAAAGAGTAATTGGCTTTCTTTGGCCTAATTAAAAGATTCCAATTGCAATACTCGTTCATAAAAAATCGTAAAAAATGCAAAGAAGAGGCATCTTT

>T5541

GTATATATTTTATTTGATACAAACTCTTTTTTATAGAAGATCCGCTATGATAATGAGAAAGATTTCTGCATATACGCCCAAATAGGGCGATAATATTAGAATCTGATAAATCAGCCCAAACCGGCTTACTAATAGGGTGCCCTAATACGTTACAAAATTTTTCTTTAGCCAATGCCGCAATCAGAGGAATAATTGGAATAAAAATATCGAACTTCTTAATGGCATTATTAATTGGAAATGCATTTTCGAGGATTTGGCTCCGTACCACTGAAGGTTTCATCAGCACACGTGAAAGATAGCCAAGAAATTCGAGGAAATGATTGGATAATTGCTTTCTAGAAATCCTTCTTGATTGAAACCACAGCGAAAAATGCCATTGCCAAAAAGCGACAAGGTAACATTTCCATTTATTCATAAAATGGAACGTCCCTTTTGAAGCCAAGAGGAATCTTCTTTGATACCTAATATAATGCATGCAAGGTTTCTTGACCAACCATAGGTTCACCTGAAAATCCTTAACCTTAACAAAGACGTTCACAAGATGTTCTATTTTTCCATGGAAATCGATTCGTTCAAGAAAACCTCCAGAAGATGTTGATCGTAAATGAGAAGATTGGTTACGGAAAAAGAAGAAAGTGGATTCATATTCCCATACATGAGAATTATCTAAGAATAAGAATAACCTTAGATTTCTTTTTGAAAAAGAGTAATTGGCTTTCTTTGGCCTAATTAAAAGATTCCAATTGCAATACTCGTTCATAAAAAATCGTAAAAAATGCAAAGAAGAGGCATCTTT

>T5541

AAAGTCGAAGTATATATTTTATTTGATACAAACTCTTTTTTATAGAAGATCCGCTATGATAATGAGAAAGATTTCTGCATATACGCCCAAATAGGGCGATAATATTAGAATCTGATAAATCAGCCCAAACCGGCTTACTAATAGGGTGCCCTAATACGTTACAAAATTTTTCTTTAGCCAATGCCGCAATCAGAGGAATAATTGGAATAAAAATATCGAACTTCTTAATGGCATTATTAATTGGAAATGCATTTTCGAGGATTTGGCTCCGTACCACTGAAGGTTTCATCAGCACACGTGAAAGATAGCCAAGAAATTCGAGGAAATGATTGGATAATTGCTTTCTAGAAATCCTTCTTGATTGAAACCACAGCGAAAAATGCCATTGCCAAAAAGCGACAAGGTAACATTTCCATTTATTCATAAAATGGAACGTCCCTTTTGAAGCCAAGAGGAATCTTCTTTGATACCTAATATAATGCATGCAAGGTTTCTTGACCAACCATAGGTTCACCTGAAAATCCTTAACCTTAACAAAGACGTTCACAAGATGTTCTATTTTTCCATGGAAATCGATTCGTTCAAGAAAACCTCCAGAAGATGTTGATCGTAAATGAGAAGATTGGTTACGGAAAAAGAAGAAAGTGGATTCATATTCCCATACATGAGAATTATCTAAGAATAAGAATAACCTTAGATTTCTTTTTGAAAAAGAGTAATTGGCTTTCTTTGGCCTAATTAAAAGATTCCAATTGCAATACTCGTTCATAAAAAATCGTAAAAAATGCAAAGAAGAGGCATCTTT

>T5543

GTATATATTTTATTCGATACAAATTCTTTTTTTTTGAAGACCCGCTGTAAAAATGAGAAATATTTCTGCATATACGCACAAATCGGTTGAGAATATCAGAATCTGATGAATCCGTCCAGGTCGCTTTACTAATGGGATGCCCTAATACATTACAAAATTTATCTTTAGCCAACGACCCAATAATAGAAGAAATTGGAATTTTACTATCCAATTTGATTCTAACATTATCTATTAGAAATGAGTTTTCTAGCAGTTGACTACGTACCACTAAAGGATTTAATCGCAAACTTGACAGATAACCCAGAAATT

>T5544

AGTATATATTTTATTTGATACAAACTCTTTTTTATAGAAGATCCGCTATGATAATGAGAAAGATTTCTGCATATACGCCCAAATAGGGCGATAATATTAGAATCTGATAAATCAGCCCAAACCGGCTTACTAATAGGGTGCCCTAATACGTTACAAAATTTTTCTTTAGCCAATGCCGCAATCAGAGGAATAATTGGAATAAAAATATCGAACTTCTTAATGGCATTATTAATTGGAAATGCATTTTCGAGGATTTGGCTCCGTACCACTGAAGGTTTCATCAGCACACGTGAAAGATAGCCAAGAAATTCGAGGAAATGATTGGATAATTGCTTTCTAGAAATCCTTCTTGATTGAAACCACAGCGAAAAATGCCATTGCCAAAAAGCGACAAGGTAACATTTCCATTTATTCATAAAATGGAACGTCCCTTTTGAAGCCAAGAGGAATCTTCTTTGATACCTAATATAATGCATGCAAGGTTTCTTGACCAACCATAGGTTCACCTGAAAATCCTTAACCTTAACAAAGACGTTCACAAGATGTTCTATTTTTCCATGGAAATCGATTCGTTCAAGAAAACCTCCAGAAGATGTTGATCGTAAATGAGAAGATTGGTTACGGAAAAAGAAGAAAGTGGATTCATATTCCCATACATGAGAATTATCTAAGAATAAGAATAACCTTAGATTTCTTTTTGAAAAAGAGTAATTGGCTTTCTTTGGCCTAATTAAAAGATTCCAATTGCAATACTCGTTCATAAAAAATCGTAAAAAATGCAAAGAAGAGGCATCTTT

>T5547

GTATATATTTTATACGATACAAACTCTTTTTTATAGAAGATCCGCTATGATAATGAGAAAGATTTCTGCATATACGCCCAAATAGGTCGATAATATTAGAATCTGATAAATCAGCCCAAACTGGCTTACTAATAGGGTGGCCTAATACGTTACAAAATTTTTCTTTAGCCAATGCCGCAATCAGAGGAATAATTGGAATAAGAACATCGAACTTCTTAATGGCATTATTGATTGGAAATGCATTTTCGAGGATTTGGCTCCGTACCACTGAAGGTTTCATCAGCACACGTGAAAGATAGCCAAGAAATTCAAGGAAATGATTGGATAATTGTTTTCTAGAAATCCTTCTTGATTGAAACCACAGCGAAAAATGCCATTGCCAAAAAGTGACAAGGTAACATTTCCATTTATTCATGAAATGGAACGTCCCTTTTGAAGCCAAGAGGAATCTTCTTTGATACCTAATATAGTGCATGCAAGGTTTCTTGACCAACCATAGGTTCACCTGAAAATCCTTAACCTTAACAAAGACGTTCACAAGATTTTCTATTTTTCCATGGAAATCAATTCGTTCAAGAAAACCTCCAGAAGATGTTGATCGTAAATGAGAAGATTGGTTACGGAAAAAGAAGAAAGTGGATTCATATTCCCATACATGAGAATTATCTAAGAATAAGAATAACCTTAGATTTCTTTTTGAAGAAGAGGAATTGGCTTTCTTTGGCCTAATTAAAAGATTCCAATTGCAATACTCGTACTCGTTCATAAAAGATCGTAAAAAATGCAAAGAAGAGGCATCTT

>T5547

AAGTCGAAGTATATATTTTATACGATACAAACTCTTTTTTATAGAAGATCCGCTATGATAATGAGAAAGATTTCTGCATATACGCCCAAATAGGTCGATAATATTAGAATCTGATAAATCAGCCCAAACTGGCTTACTAATAGGGTGGCCTAATACGTTACAAAATTTTTCTTTAGCCAATGCCGCAATCAGAGGAATAATTGGAATAAGAACATCGAACTTCTTAATGGCATTATTGATTGGAAATGCATTTTCGAGGATTTGGCTCCGTACCACTGAAGGTTTCATCAGCACACGTGAAAGATAGCCAAGAAATTCAAGGAAATGATTGGATAATTGTTTTCTAGAAATCCTTCTTGATTGAAACCACAGCGAAAAATGCCATTGCCAAAAAGTGACAAGGTAACATTTCCATTTATTCATGAAATGGAACGTCCCTTTTGAAGCCAAGAGGAATCTTCTTTGATACCTAATATAGTGCATGCAAGGTTTCTTGACCAACCATAGGTTCACCTGAAAATCCTTAACCTTAACAAAGACGTTCACAAGATTTTCTATTTTTCCATGGAAATCAATTCGTTCAAGAAAACCTCCAGAAGATGTTGATCGTAAATGAGAAGATTGGTTACGGAAAAAGAAGAAAGTGGATTCATATTCCCATACATGAGAATTATCTAAGAATAAGAATAACCTTAGATTTCTTTTTGAAGAAGAGGAATTGGCTTTCTTTGGCCTAATTAAAAGATTCCAATTGCAATACTCGTACTCGTTCATAAAAGATCGTAAAAAATGCAAAGAAGAGGCATCTTTTAACC

>T5549

GTATATATTTTATTTGATACAAACTCTTTTTTATAGAAGATCCGCTATGATAATGAGAAAGATTTCTGCATATACGCCCAAATAGGGCGATAATATTAGAATCTGATAAATCAGCCCAAACCGGCTTACTAATAGGGTGCCCTAATACGTTACAAAATTTTTCTTTAGCCAATGCCGCAATCAGAGGAATAATTGGAATAAAAATATCGAACTTCTTAATGGCATTATTAATTGGAAATGCATTTTCGAGGATTTGGCTCCGTACCACTGAAGGTTTCATCAGCACACGTGAAAGATAGCCAAGAAATTCGAGGAAATGATTGGATAATTGCTTTCTAGAAATCCTTCTTGATTGAAACCACAGCGAAAAATGCCATTGCCAAAAAGCGACAAGGTAACATTTCCATTTATTCATAAAATGGAACGTCCCTTTTGAAGCCAAGAGGAATCTTCTTTGATACCTAATATAATGCATGCAAGGTTTCTTGACCAACCATAGGTTCACCTGAAAATCCTTAACCTTAACAAAGACGTTCACAAGATGTTCTATTTTTCCATGGAAATCGATTCGTTCAAGAAAACCTCCAGAAGATGTTGATCGTAAATGAGAAGATTGGTTACGGAAAAAGAAGAAAGTGGATTCATATTCCCATACATGAGAATTATCTAAGAATAAGAATAACCTTAGATTTCTTTTTGAAAAAGAGTAATTGGCTTTCTTTGGCCTAATTAAAAGATTCC

>T5550

GTATATATTTTATTTGATACAAACTCTTTTTTATAGAAGATCCGCTATGATAATGAGAAAGATTTCTGCATATACGCCCAAATAGGGCGATAATATTAGAATCTGATAAATCAGCCCAAACCGGCTTACTAATAGGGTGCCCTAATACGTTACAAAATTTTTCTTTAGCCAATGCCGCAATCAGAGGAATAATGGGAATAAAAATATCGAACTTCTTAATGGCATTATTAATTGGAAATGCATTTTCGAGGATTTGGCTCCGTACCACTGAAGGTTTCATCAGCACACGTGAAAGATAGCCAAGAAATTC

>T5551

GTATATATTTTATTTGATACAAACTCTTTTTTATAGAAGATCCGCTATGATAATGAGAAAGATTTCTGCATATACGCCCAAATAGGGCGATAATATTAGAATCTGATAAATCAGCCCAAACCGGCTTACTAATAGGGTGCCCTAATACGTTACAAAATTTTTCTTTAGCCAATGCCGCAATCAGAGGAATAATTGGAATAAAAATATCGAACTTCTTAATGGCATTATTAATTGGAAATGCATTTTCGAGGATTTGGCTCCGTACCACTGAAGGTTTCATCAGCACACGTGAAAGATAGCCAAGAAATTCGAGGAAATGATTGGATAATTGCTTTCTAGAAATCCTTCTTGATTGAAACCACAGCGAAAAATGCCATTGCCAAAAAGCGACAAGGTAACATTTCCATTTATTCATAAAATGGAACGTCCCTTTTGAAGCCAAGAGGAATCTTCTTTGATACCTAATATAATGCATGCAAGGTTTCTTGACCAACCATAGGTTCACCTGAAAATCCTTAACCTTAACAAAGACGTTCACAAGATGTTCTATTTTTCCATGGAAATCGATTCGTTCAAGAAAACCTCCAGAAGATGTTGATCGTAAATGAGAAGATTGGTTACGGAAAAAGAAGAAAGTGGATTCATATTCCCATACATGAGAATTATCTAAGAATAAGAATAACCTTAGATTTCTTTTTGAAAAAGAGTAATTGGCTTTCTTTGGCCTAATTAAAAGATTCCAATTGCAATACTCGTTCATAAAAAATCGTAAAAA

**4c. DNA regions at the *psbA-trnH* region (primers psbAF/trnHR)**

>T5529

GCTCCAACAAATGGATAAGACTTGGTCTTAGTGTATAGGAGTTTTTCAAAATAGAATCACATAAGGAGCAATAAACTCTTTCTTGTTCTATCAAGAGAGTTTATTGCTCCTTAATTTTCTTTTCAATGACTATTGTTTTTTTAGTATTATTGTCCTTACTTAAACTTTTCTTCTTTTCCTGGACTGGAAAAGAAGGAGGACGTCTTCTATTCTTAGTCTTAGGGTATTCTTAGTCTTATAGTCTTAGGGGTTGATTAATGGTTGGGTACTATTCGTTCGTTCTCTATAAAATAGGAATTTTTTGTATCTATCTAACTTATCGAATTTCTTGTTAAATAAGTTTTTTTATTCTTTTAAAGAAATATCTTAGAAAAAAGAAAGAAAATTCTAAAAAGGTCGAAAATTTGAAGTTGAAGTTAATAATTCATTAAAATGAAAAGTCAATTTAAAT

>T5529

GCTCCAACAAATGGATAAGACTTGGTCTTAGTGTATAGGAGTTTTTCAAAATAGAATCACATAAGGAGCAATAAACTCTTTCTTGTTCTATCAAGAGAGTTTATTGCTCCTTAATTTTCTTTTCAATGACTATTGTTTTTTTAGTATTATTGTCCTTACTTAAACTTTTCTTCTTTTCCTGGACTGGAAAAGAAGGAGGACGTCTTCTATTCTTAGTCTTAGGGTATTCTTAGTCTTATAGTCTTAGGGGTTGATTAATGGTTGGGTACTATTCGTTCGTTCTCTATAAAATAGGAATTTTTTGTATCTATCTAACTTATCGAATTTCTTGTTAAATAAGTTTTTTTATTCTTTTAAAGAAATATCTTAGAAAAAAGAAAGAAAATTCTAAAAAGGTCGAAAATTTGAAGTTGAAGTTAATAATTCATTAAAATGAAAAGTCAATTTAAAT

>T5530

TAGCTGCTATCGAAGCTCCAACAAATGGATAAGACTTGGTCTTAGTGTATAGGAGTTTTTCAAAATAGAATCACATAAGGAGCAATAAACTCTTTCTTGTTCTATCAAGAGAGTTTATTGCTCCTTAATTTTCTTTTCAATGACTATTGTTTTTTTAGTATTATTGTCCTTACTTAAACTTTTCTTCTTTTCCTGGACTGGAAAAGAAGGAGGACGTCTTCTATTCTTAGTCTTAGGGTATTCTTAGTCTTATAGTCTTAGGGGTTGATTAATGGTTGGGTACTATTCGTTCGTTCTCTATAAAATAGGAATTTTTTGTATCTATCTAACTTATCGAATTTCTTGTTAAATAAGTTTTTTTATTCTTTTAAAGAAATATCTTA

>T5531

TGTATAGGAGTTTTTCAAAATAGAATCACATAAGGAGCAATAAACTCTTTCTTGTTCTATCAAGAGAGTTTATTGCTCCTTAATTTTCTTTTCAATGACTATTGTTTTTTTA

>T5532

GCTCCAACAAATGGATAAGACTTGGTCTTAGTGTATAGGAGTTTTTCAAAATAGAATCACATAAGGAGCAATAAACTCTTTCTTGTTCTATCAAGAGAGTTTATTGCTCCTTAATTTTCTTTTCAATGACTATTGTTTTTTTAGTATTATTGTCCTTACTTAAACTTTTCTTCTTTTCCTGGACTGGAAAAGAAGGAGGACGTCTTCTATTCTTAGTCTTAGGGTATTCTTAGTCTTATAGTCTTAGGGGTTGATTAATGGTTGGGTACTATTCGTTCGTTCTCTATAAAATAGGAATTTTTTGTATCTATCTAACTTATCGAATTTCTTGTTAAATAAGTTTTTTTATTCTTTTAAAGAAATATCTTAGAAAAAAGAAAGAAAATTCTAAAAAGGTCGAAAATTTGAAGTTGAAGTTAATAATTCATTAAAATGAAAAGTCAATTTAAATTACAGGGGCGGA

>T5532

TTAGCTGCTATCGAAGCTCCAACAAATGGATAAGACTTGGTCTTAGTGTATAGGAGTTTTTCAAAATAGAATCACATAAGGAGCAATAAACTCTTTCTTGTTCTATCAAGAGAGTTTATTGCTCCTTAATTTTCTTTTCAATGACTATTGTTTTTTTAGTATTATTGTCCTTACTTAAACTTTTCTTCTTTTCCTGGACTGGAAAAGAAGGAGGACGTCTTCTATTCTTAGTCTTAGGGTATTCTTAGTCTTATAGTCTTAGGGGTTGATTAATGGTTGGGTACTATTCGTTCGTTCTCTATAAAATAGGAATTTTTTGTATCTATCTAACTTATCGAATTTCTTGTTAAATAAGTTTTTTTATTCTTTTAAAGAAATATCTTAGAAAAAAGAAAGAAAATTCTAAAAAGGTCGAAAATTTGAAGTTGAAGTTAATAATTCATTAAAATGAAAAGTCAATTTAAATTACAGGGGCGGA

>T5533

TTAGCTGCTATCGAAGCTCCAACAAATGGATAAGACTTGGTCTTAGTGTATAGGAGTTTTTCAAAATAGAATCACATAAGGAGCAATAAACTCTTTCTTGTTCTATCAAGAGAGTTTATTGCTCCTTAATTTTCTTTTCAATGACTATTGTTTTTTTAGTATTATTGTCCTTACTTAAACTTTTCTTCTTTTCCTGGACTGGAAAAGAAGGAGGACGTCTTCTATTCTTAGTCTTAGGGTATTCTTAGTCTTATAGTCTTAGGGGTTGATTAATGGTTGGGTACTATTCGTTCGTTCTCTATAAAATAGGAATTTTTTGTATCTATCTAACTTATCGAATTTCTTGTTAAATAAGTTTTTTTATTCTTTTAAAGAAATATCTTAGAAAAAAGAAAGAAAATTCTAAAAAGGTCGAAAATTTGAAGTTGAAGTTAATAATTCATTAAAATGAAAAGTCAATTTAAAT

>T5533

CAAATGGATAAGACTTGGTCTTAGTGTATAGGAGTTTTTCAAAATAGAATCACATAAGGAGCAATAAACTCTTTCTTGTTCTATCAAGAGAGTTTATTGCTCCTTAATTTTCTTTTCAATGACTATTGTTTTTTTAGTATTATTGTCCTTACTTAAACTTTTCTTCTTTTCCTGGACTGGAAAAGAAGGAGGACGTCTTCTATTCTTAGTCTTAGGGTATTCTTAGTCTTATAGTCTTAGGGGTTGATTAATGGTTGGGTACTATTCGTTCGTTCTCTATAAAATAGGAATTTTTTGTATCTATCTAACTTATCGAATTTCTTGTTAAATAAGTTTTTTTATTCTTTTAAAGAAATATCTTAGAAAAAAGAAAGAAAATTCTAAAAAGGTCGAAAATTTGAAGTTGAAGTTAATAATTCATTAAAATGAAAAGTCAATTTAAATTACAGGG

>T5535

GCTCCAACAAATGGATAAGACTTGGTCTTAGTGTATAGGAGTTTTTCAAAATAGAATCACATAAGGAGCAATAAACTCTTTCTTGTTCTATCAAGAGAGTTTATTGCTCCTTAATTTTCTTTTCAATGACTATTGTTTTTTTAGTATTATTGTCCTTACTTAAACTTTTCTTCTTTTCCTGGACTGGAAAAGAAGGAGGACGTCTTCTATTCTTAGTCTTAGGGTATTCTTAGTCTTATAGTCTTAGGGGTTGATTAATGGTTGGGTACTATTCGTTCGTTCTCTATAAAATAGGAATTTTTTGTATCTATCTAACTTATCGAATTTCTTGTTAAATAAGTTTTTTTATTCTTTTAAAGAAATATCTTAGAAAAAAGAAAGAAAATTCTAAAAAGGTCGAAAATTTGAAGTTGAAGTTAATAATTCATTAAAATGAAAAGTCAATTTAAAT

>T5536

GCTCCAACAAATGGATAAGACTTGGTCTTAGTGTATAGGAGTTTTTCAAAATAGAATCACATAAGGAGCAATAAACTCTTTCTTGTTCTATCAAGAGAGTTTATTGCTCCTTAATTTTCTTTTCAATGACTATTGTTTTTTTAGTATTATTGTCCTTACTTAAACTTTTCTTCTTTTCCTGGACTGGAAAAGAAGGAGGACGTCTTCTATTCTTAGTCTTAGGGTATTCTTAGTCTTATAGTCTTAGGGGTTGATTAATGGTTGGGTACTATTCGTTCGTTCTCTATAAAATAGGAATTTTTTGTATCTATCTAACTTATCGAATTTCTTGTTAAATAAGTTTTTTTATTCTTTTAAAGAAATATCTTAGAAAAAAGAAAGAAAATTCTAAAAAGGTCGAAAATTTGAAGTTGAAGTTAATAATTCATTAAAATGAAAAGTCAATT

>T5536

GCTCCAACAAATGGATAAGACTTGGTCTTAGTGTATAGGAGTTTTTCAAAATAGAATCACATAAGGAGCAATAAACTCTTTCTTGTTCTATCAAGAGAGTTTATTGCTCCTTAATTTTCTTTTCAATGACTATTGTTTTTTTAGTATTATTGTCCTTACTTAAACTTTTCTTCTTTTCCTGGACTGGAAAAGAAGGAGGACGTCTTCTATTCTTAGTCTTAGGGTATTCTTAGTCTTATAGTCTTAGGGGTTGATTAATGGTTGGGTACTATTCGTTCGTTCTCTATAAAATAGGAATTTTTTGTATCTATCTAACTTATCGAATTTCTTGTTAAATAAGTTTTTTTATTCTTTTAAAGAAATATCTTAGAAAAAAGAAAGAAAATTCTAAAAAGGTCGAAAATTTGAAGTTGAAGTTAATAATTCATTAAAATGAAAAGTCAATTTAAAT

>T5538

TTAGCTGCTATCGAAGCTCCAACAAATGGATAAGACTTGGTCTTAGTGTATAGGAGTTTTTCAAAATAGAATCACATAAGGAGCAATAAACTCTTTCTTGTTCTATCAAGAGAGTTTATTGCTCCTTAATTTTCTTTTCAATGACTATTGTTTTTTTAGTATTATTGTCCTTACTTAAACTTTTCTTCTTTTCCTGGACTGGAAAAGAAGGAGGACGTCTTCTATTCTTAGTCTTAGGGTATTCTTAGTCTTATAGTCTTAGGGGTTGATTAATGGTTGGGTACTATTCGTTCGTTCTCTATAAAATAGGAATTTTTTGTATCTATCTAACTTATCGAATTTCTTGTTAAATAAGTTTTTTTATTCTTTTAAAGAAATATCTTAGAAAAAAGAAAGAAAATTCTAAAAAGGTCGAAAATTTGAAGTTGAAGTTAATAATTCATTAAAATGAAAAGTCAATTTAAAT

>T5538

CAAATGGATAAGACTTGGTCTTAGTGTATAGGAGTTTTTCAAAATAGAATCACATAAGGAGCAATAAACTCTTTCTTGTTCTATCAAGAGAGTTTATTGCTCCTTAATTTTCTTTTCAATGACTATTGTTTTTTTAGTATTATTGTCCTTACTTAAACTTTTCTTCTTTTCCTGGACTGGAAAAGAAGGAGGACGTCTTCTATTCTTAGTCTTAGGGTATTCTTAGTCTTATAGTCTTAGGGGTTGATTAATGGTTGGGTACTATTCGTTCGTTCTCTATAAAATAGGAATTTTTTGTATCTATCTAACTTATCGAATTTCTTGTTAAATAAGTTTTTTTATTCTTTTAAAGAAATATCTTAGAAAAAAGAAAGAAAATTCTAAAAAGGTCGAAAATTTGAAGTTGAAGTTAATAATTCATTAAAATGAAAAGTCAATTTAAAT

>T5539

CAAATGGATAAGACTTGGTCTTAGTGTATAGGAGTTTTTCAAAATAGAATCACATAAGGAGCAATAAACTCTTTCTTGTTCTATCAAGAGAGTTTATTGCTCCTTAATTTTCTTTTCAATGACTATTGTTTTTTTAGTATTATTGTCCTTACTTAAACTTTTCTTCTTTTCCTGGACTGGAAAAGAAGGAGGACGTCTTCTATTCTTAGTCTTAGGGTATTCTTAGTCTTATAGTCTTAGGGGTTGATTAATGGTTGGGTACTATTCGTTCGTTCTCTATAAAATAGGAATTTTTTGTATCTATCTAACTTATCGAATTTCTTGTTAAATAAGTTTTTTTATTCTTTTAAAGAAATATCTTAGAAAAAAGAAAGAAAATTCTAAAAAGGTCGAAAATTTGAAGTTGAAGTTAATAATTCATTAAAATGAAAAGTCAATTTAAAT

>T5539

TTAGCTGCTATCGAAGCTCCAACAAATGGATAAGACTTGGTCTTAGTGTATAGGAGTTTTTCAAAATAGAATCACATAAGGAGCAATAAACTCTTTCTTGTTCTATCAAGAGAGTTTATTGCTCCTTAATTTTCTTTTCAATGACTATTGTTTTTTTAGTATTATTGTCCTTACTTAAACTTTTCTTCTTTTCCTGGACTGGAAAAGAAGGAGGACGTCTTCTATTCTTAGTCTTAGGGTATTCTTAGTCTTATAGTCTTAGGGGTTGATTAATGGTTGGGTACTATTCGTTCGTTCTCTATAAAATAGGAATTTTTTGTATCTATCTAACTTATCGAATTTCTTGTTAAATAAGTTTTTTTATTCTTTTAAAGAAATATCTTAGAAAAAAGAAAGAAAATTCTAAAAAGGTCGAAAATTTGAAGTTGAAGTTAATAATTCATTAAAATGAAAAGTCAATT

>T5540

GCTCCAACAAATGGATAAGACTTGGTCTTAGTGTATAGGAGTTTTTCAAAATAGAATCACATAAGGAGCAATAAACTCTTTCTTGTTCTATCAAGAGAGTTTATTGCTCCTTAATTTTCTTTTCAATGACTATTGTTTTTTTAGTATTATTGTCCTTACTTAAACTTTTCTTCTTTTCCTGGACTGGAAAAGAAGGAGGACGTCTTCTATTCTTAGTCTTAGGGTATTCTTAGTCTTATAGTCTTAGGGGTTGATTAATGGTTGGGTACTATTCGTTCGTTCTCTATAAAATAGGAATTTTTTGTATCTATCTAACTTATCGAATTTCTTGTTAAATAAGTTTTTTTATTCTTTTAAAGAAATATCTTAGAAAAAAGAAAGAAAATTCTAAAAAGGTCGAAAATTTGAAGTTGAAGTTAATAATTCATTAAAATGAAAAGTCAATTTAAAT

>T5541

GCTCCAACAAATGGATAAGACTTGGTCTTAGTGTATAGGAGTTTTTCAAAATAGAATCACATAAGGAGCAATAAACTCTTTCTTGTTCTATCAAGAGAGTTTATTGCTCCTTAATTTTCTTTTCAATGACTATTGTTTTTTTAGTATTATTGTCCTTACTTAAACTTTTCTTCTTTTCCTGGACTGGAAAAGAAGGAGGACGTCTTCTATTCTTAGTCTTAGGGTATTCTTAGTCTTATAGTCTTAGGGGTTGATTAATGGTTGGGTACTATTCGTTCGTTCTCTATAAAATAGGAATTTTTTGTATCTATCTAACTTATCGAATTTCTTGTTAAATAAGTTTTTTTATTCTTTTAAAGAAATATCTTAGAAAAAAGAAAGAAAATTCTAAAAAGGTCGAAAATTTGAAGTTGAAGTTAATAATTCATTAAAATGAAAAGTCAATTTAAAT

>T5542

TTAGCTGCTATCGAAGCTCCAACAAATGGATAAGACTTGGTCTTAGTGTATAGGAGTTTTTCAAAATAGAATCACATAAGGAGCAATAAACTCTTTCTTGTTCTATCAAGAGAGTTTATTGCTCCTTAATTTTCTTTTCAATGACTATTGTTTTTTTAGTATTATTGTCCTTACTTAAACTTTTCTTCTTTTCCTGGACTGGAAAAGAAGGAGGACGTCTTCTATTCTTAGTCTTAGGGTATTCTTAGTCTTATAGTCTTAGGGGTTGATTAATGGTTGGGTACTATTCGTTCGTTCTCTATAAAATAGGAATTTTTTGTATCTATCTAACTTATCGAATTTCTTGTTAAATAAGTTTTTTTATTCTTTTAAAGAAATATCTTAGAAAAAAGAAAGAAAATTCTAAAAAGGTCGAAAATTTGAAGTTGAAGTTAATAATTCATTAAAATGAAAAGTCAATTTAAAT

>T5543

CTAGCTGCTGTTGAGGCTCCATCTACAAATGGATAATTCTTTAGCGTTAGTATAGACCCAGTTTAGTAATATTAAAAAACGAGCAATATAAGTCTTCGGGGAACTGTAAATTCCTACTTAAGAAGGCTTATATTGCTCGTTTTTTATAGAAAAAAATGCTCCAATCTTTTGAGCAATATAAACCTTCGGGGAACTGTAAATTCCTACTTACAGAAGGCTTATATTGCGCATTTTTATAGAAAAAGATAAGTAACTATTTCGAGCAATATAAACCTTCGGGGGACTGTAAATTCCTACTTTACAAGGTTTATATTGTGTTTTTATATTGTGTTTTTATATTGTGTTTTTTTATTTTTTTATTGTGTTTTTTTATTGTGTTTTTTTATAGAAAAAAGATGAGTAACTATTTCGAGCAATATAAACCTTCGGGGAACTGTAAATTCCTACTTTAGAAGGTTTATATTGCTCATTTTTTTCTATAAAAATGAGTAAATATTTTTGGAGTATCATATTTTTCTATTTTTTTAATAGAAAAAAGGTACTACTCAATTTTTTTTAGAAAAAAAGTAAGGTGGAATTTGCTACCAGTTTTTTTATTGAAATCTATCCGTTTTTATTTTAGACAATACAAAAAAGAATAGTAGAGTGGGGGCGGA

>T5544

TTAGCTGCTATCGAAGCTCCAACAAATGGATAAGACTTGGTCTTAGTGTATAGGAGTTTTTCAAAATAGAATCACATAAGGAGCAATAAACTCTTTCTTGTTCTATCAAGAGAGTTTATTGCTCCTTAATTTTCTTTTCAATGACTATTGTTTTTTTAGTATTATTGTCCTTACTTAAACTTTTCTTCTTTTCCTGGACTGGAAAAGAAGGAGGACGTCTTCTATTCTTAGTCTTAGGGTATTCTTAGTCTTATAGTCTTAGGGGTTGATTAATGGTTGGGTACTATTCGTTCGTTCTCTATAAAATAGGAATTTTTTGTATCTATCTAACTTATCGAATTTCTTGTTAAATAAGTTTTTTTATTCTTTTAAAGAAATATCTTAGAAAAAAGAAAGAAAATTCTAAAAAGGTCGAAAATTTGAAGTTGAAGTTAATAATTCATTAAAATGAAAAGTCAATTTAAATTACAGGGGCGGA

>T5544

TTAGCTGCTATCGAAGCTCCAACAAATGGATAAGACTTGGTCTTAGTGTATAGGAGTTTTTCAAAATAGAATCACATAAGGAGCAATAAACTCTTTCTTGTTCTATCAAGAGAGTTTATTGCTCCTTAATTTTCTTTTCAATGACTATTGTTTTTTTAGTATTATTGTCCTTACTTAAACTTTTCTTCTTTTCCTGGACTGGAAAAGAAGGAGGACGTCTTCTATTCTTAGTCTTAGGGTATTCTTAGTCTTATAGTCTTAGGGGTTGATTAATGGTTGGGTACTATTCGTTCGTTCTCTATAAAATAGGAATTTTTTGTATCTATCTAACTTATCGAATTTCTTGTTAAATAAGTTTTTTTATTCTTTTAAAGAAATATCTTAGAAAAAAGAAAGAAAATTCTAAAAAGGTCGAAAATTTGAAGTTGAAGTTAATAATTCATTAAAATGAAAAGTCAATTTAAAT

>T5547

TTTAGCTGCTATCGAAGCTCCAACAAATGGATAAGACTTGGTCTTAGTGTATAGGAGTTTTTCAAAATAGAATCACATAAGGAGCAATAAACTCTTTCTTGTTCTATCAAGAGAGTTTATTGCTCCTTAATTTTCTTTTCAATGACTATTGTTTTTTTAGTATTATTGTCCTTACTTAAACTTTTCTTCTTTTCCTGGACTGGAAAAGAAGGAGGACGTCTTCTATTCTTAGTCTTAGGGTATTCTTAGTCTTATAGTCTTAGGGGTTGATTAATGGTTGGGTACTATTCGTTCGTTCTCTATAAAATAGGAATTTTTTGTATCTATCTAACTTATCGAATTTCTTGTTAAATAAGTTTTTTATTCTTTTAAAGAAATATCTTAGAAAAAAGAAAGAAAATTCGAAAAAGGTCGAAAATTTGAAGTTGAAGTTAATAATTCATTAAAATGAAAAGTCAATTTAAATTACAGGGGCGGA

>T5547

TCGAAGCTCCAACAAATGGATAAGACTTGGTCTTAGTGTATAGGAGTTTTTCAAAATAGAATCACATAAGGAGCAATAAACTCTTTCTTGTTCTATCAAGAGAGTTTATTGCTCCTTAATTTTCTTTTCAATGACTATTGTTTTTTTAGTATTATTGTCCTTACTTAAACTTTTCTTCTTTTCCTGGACTGGAAAAGAAGGAGGACGTCTTCTATTCTTAGTCTTAGGGTATTCTTAGTCTTATAGTCTTAGGGGTTGATTAATGGTTGGGTACTATTCGTTCGTTCTCTATAAAATAGGAATTTTTTGTATCTATCTAACTTATCGAATTTCTTGTTAAATAAGTTTTTTATTCTTTTAAAGAAATATCTTAGAAAAAAGAAAGAAAATTCGAAAAAGGTCGAAAATTTGAAGTTGAAGTTAATAATTCATTAAAATGAAAAGTCAATTTAAATTACAGGGGCGGATG

>T5549

GCTCCAACAAATGGATAAGACTTGGTCTTAGTGTATAGGAGTTTTTCAAAATAGAATCACATAAGGAGCAATAAACTCTTTCTTGTTCTATCAAGAGAGTTTATTGCTCCTTAATTTTCTTTTCAATGACTATTGTTTTTTTAGTATTATTGTCCTTACTTAAACTTTTCTTCTTTTCCTGGACTGGAAAAGAAGGAGGACGTCTTCTATTCTTAGTCTTAGGGTATTCTTAGTCTTATAGTCTTAGGGGTTGATTAATGGTTGGGTACTATTCGTTCGTTCTCTATAAAATAGGAATTTTTTGTATCTATCTAACTTATCGAATTTCTTGTTAAATAAGTTTTTTTATTCTTTTAAAGAAATATCTTAGAAAAAAGAAAGAAAATTCTAAAAAGGTCGAAAATTTGAAGTTGAAGTTAATAATTCATTAAAATGAAAAGTCAATTTAAATTACAGGGGCGGAT

>T5549

GCTCCAACAAATGGATAAGACTTGGTCTTAGTGTATAGGAGTTTTTCAAAATAGAATCACATAAGGAGCAATAAACTCTTTCTTGTTCTATCAAGAGAGTTTATTGCTCCTTAATTTTCTTTTCAATGACTATTGTTTTTTTAGTATTATTGTCCTTACTTAAACTTTTCTTCTTTTCCTGGACTGGAAAAGAAGGAGGACGTCTTCTATTCTTAGTCTTAGGGTATTCTTAGTCTTATAGTCTTAGGGGTTGATTAATGGTTGGGTACTATTCGTTCGTTCTCTATAAAATAGGAATTTTTTGTATCTATCTAACTTATCGAATTTCTTGTTAAATAAGTTTTTTTATTCTTTTAAAGAAATATCTTAGAAAAAAGAAAGAAAATTCTAAAAAGGTCGAAAATTTGAAGTTGAAGTTAATAATTCATTAAAATGAAAAGTCAATTTAAAT

>T5549

GCTCCAACAAATGGATAAGACTTGGTCTTAGTGTATAGGAGTTTTTCAAAATAGAATCACATAAGGAGCAATAAACTCTTTCTTGTTCTATCAAGAGAGTTTATTGCTCCTTAATTTTCTTTTCAATGACTATTGTTTTTTTAGTATTATTGTCCTTACTTAAACTTTTCTTCTTTTCCTGGACTGGAAAAGAAGGAGGACGTCTTCTATTCTTAGTCTTAGGGTATTCTTAGTCTTATAGTCTTAGGGGTTGATTAATGGTTGGGTACTATTCGTTCGTTCTCTATAAAATAGGAATTTTTTGTATCTATCTAACTTATCGAATTTCTTGTTAAATAAGTTTTTTTATTCTTTTAAAGAAATATCTTAGAAAAAAGAAAGAAAATTCTAAAAAGGTCGAAAATTTGAAGTTGAAGTTAATAATTCATTAAAATGAAAAGTCAATTTAAAT

>T5550

TTAGCTGCTATCGAAGCTCCAACAAATGGATAAGACTTGGTCTTAGTGTATAGGAGTTTTTCAAAATAGAATCACATAAGGAGCAATAAACTCTTTCTTGTTCTATCAAGAGAGTTTATTGCTCCTTAATTTTCTTTTCAATGACTATTGTTTTTTTAGTATTATTGTCCTTACTTAAACTTTTCTTCTTTTCCTGGACTGGAAAAGAAGGAGGACGTCTTCTATTCTTAGTCTTAGGGTATTCTTAGTCTTATAGTCTTAGGGGTTGATTAATGGTTGGGTACTATTCGTTCGTTCTCTATAAAATAGGAATTTTTTGTATCTATCTAACTTATCGAATTTCTTGTTAAATAAGTTTTTTTATTCTTTTAAAGAAATATCTTA

>T5551

GCTCCAACAAATGGATAAGACTTGGTCTTAGTGTATAGGAGTTTTTCAAAATAGAATCACATAAGGAGCAATAAACTCTTTCTTGTTCTATCAAGAGAGTTTATTGCTCCTTAATTTTCTTTTCAATGACTATTGTTTTTTTAGTATTATTGTCCTTACTTAAACTTTTCTTCTTTTCCTGGACTGGAAAAGAAGGAGGACGTCTTCTATTCTTAGTCTTAGGGTATTCTTAGTCTTATAGTCTTAGGGGTTGATTAATGGTTGGGTACTATTCGTTCGTTCTCTATAAAATAGGAATTTTTTGTATCTATCTAACTTATCGAATTTCTTGTTAAATAAGTTTTTTTATTCTTTTAAAGAAATATCTTAGAAAAAAGAAAGAAAATTCTAAAAAGGTCGAAAATTTGAAGTTGAAGTTAATAATTCATTAAAATGAAAAGTCAATTTAAATTACAGGGGCGGA

**4d. DNA regions at the *rbcL* region (primers rbcLaF/rbcLaR)**

>T5529

GGGTGTTAAAGAGTACAAATTGACTTATTATACTCCTGAATATGAAACCAAAGATACTGATATCTTGGCAGCATTCCGAGTAACTCCTCAACCGGGAGTTCCACCTGAAGAAGCAGGGGCCGCGGTAGCTGCCGAATCCTCCACCGGTACATGGACAACCGTGTGGACCGATGGACTTACCAGCCTTGATCGTTACAAAGGGCGATGCTACAACATCGAGGCCGTTCCTGGCGAAGCAGATCAATACATCTGTTATGTAGCTTACCCTTTAGACCTTTTTGAAGAAGGTTCTGTTACCAACATGTTTACTTCCATTGTAGGAAATGTTTTTGGATTCAAAGCACTGCGTGCTCTACGTCTGGAAGATCTGCGAATCCCTGTTGCTTATGTTAAAACTTTCCAGGGCCCGCCTCATGGGATCCAAAGTGAGAGAGATAAATTGAACAAGTATGGTCGTCCTCTGCTGGGATGTACTATTAAACCTAAATTGGGGTTATCCGCTAAAAACTAT

>T5530

GAGTACAAATTGACTTATTATACTCCTGAATATGAAACCAAAGATACTGATATCTTGGCAGCATTCCGAGTAACTCCTCAACCGGGAGTTCCACCCGAAGAAGCAGGGGCCGCGGTAGCTGCCGAATCCTCCACCGGTACATGGACAACCGTGTGGACCGATGGACTTACCAGCCTTGATCGTTACAAAGGGCGATGCTACAACATCGAGGCCGTTCCTGGCGAAGCAGATCAATACATCTGTTATGTAGCTTACCCTTTAGACCTTTTTGAAGAAGGTTCTGTTACCAACATGTTTACTTCCATTGTAGGAAATGTTTTTGGATTCAAAGCACTGCGTGCTCTACGTCTGGAAGATCTGCGAATCCCTGTTGCTTATGTTAAAACTTTCCAGGGCCCGCCTCATGGGATCCAAAGTGAGAGAGATAAATTGAACAAGTATGGTCGTCCTCTGCTGGGATGTACTATTAAACCTAAATTGGGGTTATCCGCTAAA

>T5530

GAGTACAAATTGACTTATTATACTCCTGAATATGAAACCAAAGATACTGATATCTTGGCAGCATTCCGAGTAACTCCTCAACCGGGAGTTCCACCCGAAGAAGCAGGGGCCGCGGTAGCTGCCGAATCCTCCACCGGTACATGGACAACCGTGTGGACCGATGGACTTACCAGCCTTGATCGTTACAAAGGGCGATGCTACAACATCGAGGCCGTTCCTGGCGAAGCAGATCAATACATCTGTTATGTAGCTTACCCTTTAGACCTTTTTGAAGAAGGTTCTGTTACCAACATGTTTACTTCCATTGTAGGAAATGTTTTTGGATTCAAAGCACTGCGTGCTCTACGTCTGGAAGATCTGCGAATCCCTGTTGCTTATGTTAAAACTTTCCAGGGCCCGCCTCATGGGATCCAAAGTGAGAGAGATAAATTGAACAAGTATGGTCGTCCTCTGCTGGGATGTACTATTAAACCTAAATTGGGGTTATCCGCTAAA

>T5531

GAGTACAAATTGACTTATTATACTCCTGAATATGAAACCAAAGATACTGATATCTTGGCAGCATTCCGAGTAACTCCTCAACCGGGAGTTCCACCTGAAGAAGCAGGGGCCGCGGTAGCTGCCGAATCCTCCACCGGTACATGGACAACCGTGTGGACCGATGGACTTACCAGCCTTGATCGTTACAAAGGGCGATGCTACAACATCGAGCCCGTTCCTGGCGAAGCAGATCAATACATCTGTTATGTAGCTTACCCTTTAGACCTTTTTGAAGAAGGTTCTGTTACCAACATGTTTACTTCCATTGTAGGAAATGTTTTTGGATTCAAAGCACTGCGTGCTCTACGTCTGGAAGATCTGCGAATCCCTGTTGCTTATGTTAAAACTTTCCAGGGTCCGCCTCATGGGATCCAAAGTGAGAGAGATAAATTGAACAAGTATGGTCGTCCTCTGCTGGGATGTACTATTAAACCTAAATTGGGGTTATCCGCTAAAAACTATG

>T5532

GAGTACAAATTGACTTATTATACTCCTGAATATGAAACCAAAGATACTGATATCTTGGCAGCATTCCGAGTAACTCCTCAACCGGGAGTTCCACCTGAAGAAGCAGGGGCCGCGGTAGCTGCCGAATCCTCCACCGGTACATGGACAACCGTGTGGACCGATGGACTTACCAGCCTTGATCGTTACAAAGGGCGATGCTACAACATCGAGGCCGTTCCTGGCGAAGCAGATCAATACATCTGTTATGTAGCTTACCCTTTAGACCTTTTTGAAGAAGGTTCTGTTACCAACATGTTTACTTCCATTGTAGGAAATGTTTTTGGATTCAAAGCACTGCGTGCTCTACGTCTGGAAGATCTGCGAATCCCTGTTGCTTATGTTAAAACTTTCCAGGGCCCGCCTCATGGGATCCAAAGTGAGAGAGATAAATTGAACAAGTATGGTCGTCCTCTGCTGGGATGTACTATTAAACCTAAATTGGGGTTATCCGCTAAAA

>T5532

GAGTACAAATTGACTTATTATACTCCTGAATATGAAACCAAAGATACTGATATCTTGGCAGCATTCCGAGTAACTCCTCAACCGGGAGTTCCACCTGAAGAAGCAGGGGCCGCGGTAGCTGCCGAATCCTCCACCGGTACATGGACAACCGTGTGGACCGATGGACTTACCAGCCTTGATCGTTACAAAGGGCGATGCTACAACATCGAGGCCGTTCCTGGCGAAGCAGATCAATACATCTGTTATGTAGCTTACCCTTTAGACCTTTTTGAAGAAGGTTCTGTTACCAACATGTTTACTTCCATTGTAGGAAATGTTTTTGGATTCAAAGCACTGCGTGCTCTACGTCTGGAAGATCTGCGAATCCCTGTTGCTTATGTTAAAACTTTCCAGGGCCCGCCTCATGGGATCCAAAGTGAGAGAGATAAATTGAACAAGTATGGTCGTCCTCTGCTGGGATGTACTATTAAACCTAAATTGGGGTTATCCGCTAAAA

>T5533

GAGTACAAATTGACTTATTATACTCCTGAATATGAAACCAAAGATACTGATATCTTGGCAGCATTCCGAGTAACTCCTCAACCGGGAGTTCCACCTGAAGAAGCAGGGGCCGCGGTAGCTGCCGAATCCTCCACCGGTACATGGACAACCGTGTGGACCGATGGACTTACCAGCCTTGATCGTTACAAAGGGCGATGCTACAACATCGAGGCCGTTCCTGGCGAAGCAGATCAATACATCTGTTATGTAGCTTACCCTTTAGACCTTTTTGAAGAAGGTTCTGTTACCAACATGTTTACTTCCATTGTAGGAAATGTTTTTGGATTCAAAGCACTGCGTGCTCTACGTCTGGAAGATCTGCGAATCCCTGTTGCTTATGTTAAAACTTTCCAGGGCCCGCCTCATGGGATCCAAAGTGAGAGAGATAAATTGAACAAGTATGGTCGTCCTCTGCTGGGATGTACTATTAAACCTAAATTGGGGTTATCCGCTAAAAACTAT

>T5535

GAGTACAAATTGACTTATTATACTCCTGAATATGAAACCAAAGATACTGATATCTTGGCAGCATTCCGAGTAACTCCTCAACCGGGAGTTCCACCTGAAGAAGCAGGGGCCGCGGTAGCTGCCGAATCCTCCACCGGTACATGGACAACCGTGTGGACCGATGGACTTACCAGCCTTGATCGTTACAAAGGG

>T5535

GATGCTACAACATCGAGGCCGTTCCTGGCGAAGCAGATCAATACATCTGTTATGTAGCTTACCCTTTAGACCTTTTTGAAGAAGGTTCTGTTACCAACATGTTTACTTCCATTGTAGGAAATGTTTTTGGATTCAAAGCACTGCGTGCTCTACGTCTGGAAGATCTGCGAATCCCTGTTGCTTATGTTAAAACTTTCCAGGGCCCGCCTCATGGGATCCAAAGTGAGAGAGATAAATTGAACAAGTATGGTCGTCCTCTGCTGGGATGTACTATTAAACCTAAATTGGGGTTATCCGCTAAAA

>T5536

GAGTACAAATTGACTTATTATACTCCTGAATATGAAACCAAAGATACTGATATCTTGGCAGCATTCCGAGTAACTCCTCAACCGGGAGTTCCACCTGAAGAAGCAGGGGCCGCGGTAGCTGCCGAATCCTCCACCGGTACATGGACAACCGTGTGGACCGATGGACTTACCAGCCTTGATCGTTACAAAGGGCGATGCTACAACATCGAGGCCGTTCCTGGCGAAGCAGATCAATACATCTGTTATGTAGCTTACCCTTTAGACCTTTTTGAAGAAGGTTCTGTTACCAACATGTTTACTTCCATTGTAGGAAATGTTTTTGGATTCAAAGCACTGCGTGCTCTACGTCTGGAAGATCTGCGAATCCCTGTTGCTTATGTTAAAACTTTCCAGGGCCCGCCTCATGGGATCCAAAGTGAGAGAGATAAATTGAACAAGTATGGTCGTCCTCTGCTGGGATGTACTATTAAACCTAAATTGGGGTTATCCGCTAAAAACTATG

>T5536

GAGTACAAATTGACTTATTATACTCCTGAATATGAAACCAAAGATACTGATATCTTGGCAGCATTCCGAGTAACTCCTCAACCGGGAGTTCCACCTGAAGAAGCAGGGGCCGCGGTAGCTGCCGAATCCTCCACCGGTACATGGACAACCGTGTGGACCGATGGACTTACCAGCCTTGATCGTTACAAAGGGCGATGCTACAACATCGAGGCCGTTCCTGGCGAAGCAGATCAATACATCTGTTATGTAGCTTACCCTTTAGACCTTTTTGAAGAAGGTTCTGTTACCAACATGTTTACTTCCATTGTAGGAAATGTTTTTGGATTCAAAGCACTGCGTGCTCTACGTCTGGAAGATCTGCGAATCCCTGTTGCTTATGTTAAAACTTTCCAGGGCCCGCCTCATGGGATCCAAAGTGAGAGAGATAAATTGAACAAGTATGGTCGTCCTCTGCTGGGATGTACTATTAAACCTAAATTGGGGTTATCCGCTAAAAACTATG

>T5537

GATTATAAATTGACTTATTATACTCCTGACTATAAAACCAAGGATACTGATATCTTGGCAGCATTTCGAGTAACTCCTCAACCCGGAGTTCCGCCTGAAGAAGCAGGGGCCGCAGTAGCTGCCGAATCTTC

>T5537

ACTGGTACATGGACAACTGTGTGGACCGATGGACTTACGAGCCTTGATCGTTACAAAGGGCGATGCTATGGAATCGAGCC

>T5537

GTTCCTGGAGAAGAGTCTCAATTTATTGCTTTTGTAGCTTACCCATTAGACCTTTTTGAAGAAGGTTCTGTTACTAACATGTTTACTTCCATTGTAGGTAATGTATTTGGGTTCAAAGCCCTGCGTGCTCTACGTCTGGAA

>T5537

ATTTGCGAATCCCTATTTCGTATGTTAAAACTTTCCAAGGTCCGCCTCACGGGATCCAAG

>T5537

TGAGAGAGATAAATTGAACAAGTATGGTCGTCCCCTGTTGGGATGTACTATTAAACCTAAATTGGGGTTATCCGCTAAAAACTA

>T5537

GATTATAAATTGACTTATTATACTCCTGACTATAAAACCAAGGATACTGATATCTTGGCAGCATTT

>T5537

GAGTAACTCCTCAACCCGGAGTTCCGCCTGAAGAAGCAGGGGCCGCAGTAGCTGCCGAATCTTCTA

>T5537

TGGTACATGGACAACTGTGTGGACCGATGGACTTACGAGCCTTGATCGTTACAAAGGGCGATGCTATGGAATCGAGCCTGTTCCTGGAGAAGAGTCTCAATTTATTGCTTTTGTAGCTTACCCATTAGACCTTTTTGAAGAAGGTTCTGTTACTAACATGTTTACTTCCATTGTAGGTAATGTATTTGGGTTCAAAGCCCTGCGTGCTCTACGTCTGGAAGATTTGCGAATCCCTATTTCGTATGTTAAAACTTTCCAAGGTCCGCCTCACGGGATCCAAGTTGAGAGAGATAAATTGAACAAGTATGGTCGTCCCCTGTTGGGATGTACTATTAAACCTAAATTGGGGTTATCCGCT

>T5538

GAGTACAAATTGACTTATTATACTCCTGAATATGAAACCAAAGATACTGATATCTTGGCAGCATTCCGAGTAACTCCTCAACCGGGAGTTCCACCTGAAGAAGCAGGGGCCGCGGTAGCTGCCGAATCCTCCACCGGTACATGGACAACCGTGTGGACCGATGGACTTACCAGCCTTGATCGTTACAAAGGGCGATGCTACAACATCGAGGCCGTTCCTGGCGAAGCAGATCAATACATCTGTTATGTAGCTTACCCTTTAGACCTTTTTGAAGAAGGTTCTGTTACCAACATGTTTACTTCCATTGTAGGAAATGTTTTTGGATTCAAAGCACTGCGTGCTCTACGTCTGGAAGATCTGCGAATCCCTGTTGCTTATGTTAAAACTTTCCAGGGCCCGCCTCATGGGATCCAAAGTGAGAGAGATAAATTGAACAAGTATGGTCGTCCTCTGCTGGGATGTACTATTAAACCTAAATTGGGGTTATCCGCTAAAA

>T5538

GAGTACAAATTGACTTATTATACTCCTGAATATGAAACCAAAGATACTGATATCTTGGCAGCATTCCGAGTAACTCCTCAACCGGGAGTTCCACCTGAAGAAGCAGGGGCCGCGGTAGCTGCCGAATCCTCCACCGGTACATGGACAACCGTGTGGACCGATGGACTTACCAGCCTTGATCGTTACAAAGGGCGATGCTACAACATCGAGGCCGTTCCTGGCGAAGCAGATCAATACATCTGTTATGTAGCTTACCCTTTAGACCTTTTTGAAGAAGGTTCTGTTACCAACATGTTTACTTCCATTGTAGGAAATGTTTTTGGATTCAAAGCACTGCGTGCTCTACGTCTGGAAGATCTGCGAATCCCTGTTGCTTATGTTAAAACTTTCCAGGGCCCGCCTCATGGGATCCAAAGTGAGAGAGATAAATTGAACAAGTATGGTCGTCCTCTGCTGGGATGTACTATTAAACCTAAATTGGGGTTATCCGCTAAAAACTATG

>T5539

AAGAGTACAAATTGACTTATTATACTCCTGAATATGAAACCAAAGATACTGATATCTTGGCAGCATTCCGAGTAACTCCTCAACCGGGAGTTCCACCTGAAGAAGCAGGGGCCGCGGTAGCTGCCGAATCCTCCACCGGTACATGGACAACCGTGTGGACCGATGGACTTACCAGCCTTGATCGTTACAAAGGGCGATGCTACAACATCGAGGCCGTTCCTGGCGAAGCAGATCAATACATCTGTTATGTAGCTTACCCTTTAGACCTTTTTGAAGAAGGTTCTGTTACCAACATGTTTACTTCCATTGTAGGAAATGTTTTTGGATTCAAAGCACTGCGTGCTCTACGTCTGGAAGATCTGCGAATCCCTGTTGCTTATGTTAAAACTTTCCAGGGCCCGCCTCATGGGATCCAAAGTGAGAGAGATAAATTGAACAAGTATGGTCGTCCTCTGCTGGGATGTACTATTAAACCTAAATTGGGGTTATCCGCTAAAAACTATG

>T5542

GAGTACAAATTGACTTATTATACTCCTGAATATGAAACCAAAGATACTGATATCTTGGCAGCATTCCGAGTAACTCCTCAACCGGGAGTTCCACCTGAAGAAGCAGGGGCCGCGGTAGCTGCCGAATCCTCCACCGGTACATGGACAACCGTGTGGACCGATGGACTTACCAGCCTTGATCGTTACAAAGGGCGATGCTACAACATCGAGGCCGTTCCTGGCGAAGCAGATCAATACATCTGTTATGTAGCTTACCCTTTAGACCTTTTTGAAGAAGGTTCTGTTACCAACATGTTTACTTCCATTGTAGGAAATGTTTTTGGATTCAAAGCACTGCGTGCTCTACGTCTGGAAGATCTGCGAATCCCTGTTGCTTATGTTAAAACTTTCCAGGGCCCGCCTCATGGGATCCAAAGTGAGAGAGATAAATTGAACAAGTATGGTCGTCCTCTGCTGGGATGTACTATTAAACCTAAATTGGGGTTATCCGCTAAA

>T5542

GAGTACAAATTGACTTATTATACTCCTGAATATGAAACCAAAGATACTGATATCTTGGCAGCATTCCGAGTAACTCCTCAACCGGGAGTTCCACCTGAAGAAGCAGGGGCCGCGGTAGCTGCCGAATCCTCCACCGGTACATGGACAACCGTGTGGACCGATGGACTTACCAGCCTTGATCGTTACAAAGGGCGATGCTACAACATCGAGGCCGTTCCTGGCGAAGCAGATCAATACATCTGTTATGTAGCTTACCCTTTAGACCTTTTTGAAGAAGGTTCTGTTACCAACATGTTTACTTCCATTGTAGGAAATGTTTTTGGATTCAAAGCACTGCGTGCTCTACGTCTGGAAGATCTGCGAATCCCTGTTGCTTATGTTAAAACTTTCCAGGGCCCGCCTCATGGGATCCAAAGTGAGAGAGATAAATTGAACAAGTATGGTCGTCCTCTGCTGGGATGTACTATTAAACCTAAATTGGGGTTATCCGCTAAA

>T5543

GAGTATAAATTGACTTATTATACTCCTGAATATGAAACCAAGGATACTGATATCTTGGCAGCATTCCGAGTAACTCCTCAACCCGGAGTTCCACCTGAAGAAGCAGGGGCTGCGGTAGCTGCTGAATCTTCTACTGGTACATGGACAACTGTGTGGACCGATGGGCTTACCAGCCTTGATCGTTACAAAGGAAGATGCTACCACATCGAGCCCGTTCCAGGAGAAGAAACTCAATTTATTGCGTATGTAGCTTACCCCTTAGACCTTTTTGAAGAAGGGTCGGTTACTAACATGTTTACCTCGATTGTGGGTAATGTATTTGGGTTCAAAGCCCTGGCTGCTCTACGTCTAGAGGATCTGCGAATCCCTCCTGCTTATACTAAAACTTTCCAGGGACCACCTCATGGTATCCAAGTTGAAAGAGATAAATTGAACAAGTACGGACGTCCCCTATTAGGATGTACTATTAAACCTAAATTGGGGTTATCCGCGAAGAATTATG

>T5543

GAGTATAAATTGACTTATTATACTCCTGAATATGAAACCAAGGATACTGATATCTTGGCAGCATTCCGAGTAACTCCTCAACCCGGAGTTCCACCTGAAGAAGCAGGGGCTGCGGTAGCTGCTGAATCTTCTACTGGTACATGGACAACTGTGTGGACCGATGGGCTTACCAGCCTTGATCGTTACAAAGGAAGATGCTACCACATCGAGCCCGTTCCAGGAGAAGAAACTCAATTTATTGCGTATGTAGCTTACCCCTTAGACCTTTTTGAAGAAGGGTCGGTTACTAACATGTTTACCTCGATTGTGGGTAATGTATTTGGGTTCAAAGCCCTGGCTGCTCTACGTCTAGAGGATCTGCGAATCCCTCCTGCTTATACTAAAACTTTCCAGGGACCACCTCATGGTATCCAAGTTGAAAGAGATAAATTGAACAAGTACGGACGTCCCCTATTAGGATGTACTATTAAACCTAAATTGGGGTTATCCGCGAAGAATTATG

>T5544

GAGTACAAATTGACTTATTATACTCCTGAATATGAAACCAAAGATACTGATATCTTGGCAGCATTCCGAGTAACTCCTCAACCGGGAGTTCCACCTGAAGAAGCAGGGGCCGCGGTAGCTGCCGAATCCTCCACCGGTACATGGACAACCGTGTGGACCGATGGACTTACCAGCCTTGATCGTTACAAAGGGCGATGCTACAACATCGAGGCCGTTCCTGGCGAAGCAGATCAATACATCTGTTATGTAGCTTACCCTTTAGACCTTTTTGAAGAAGGTTCTGTTACCAACATGTTTACTTCCATTGTAGGAAATGTTTTTGGATTCAAAGCACTGCGTGCTCTACGTCTGGAAGATCTGCGAATCCCTGTTGCTTATGTTAAAACTTTCCAGGGCCCGCCTCATGGGATCCAAAGTGAGAGAGATAAATTGAACAAGTACGGTCGTCCTCTGCTGGGATGTACTATTAAACCTAAATTGGGGTTATCCGCTAAAAACTATGGTAGAG

>T5547

GAGTACAAATTGACTTATTATACTCCTGAATATGAAACCAAAGATACTGATATCTTGGCAGCATTCCGAGTAACTCCTCAACCGGGAGTTCCACCTGAAGAAGCAGGGGCCGCGGTAGCTGCCGAATCCTCCACCGGTACATGGACAACCGTGTGGACCGATGGACTTACCAGCCTTGATCGTTACAAAGGGCGATGCTACAACATCGAGCCCGTTCCTGGCGAAGCAGATCAATACATCTGTTATGTAGCTTACCCTTTAGACCTTTTTGAAGAAGGTTCTGTTACCAACATGTTTACTTCCATTGTAGGAAATGTTTTTGGATTCAAAGCCCTGCGTGCTCTACGTCTGGAAGATCTGCGAATCCCTGTTGCTTATGTTAAAACTTTCCAGGGTCCGCCTCATGGGATCCAAAGTGAGAGAGATAAATTGAACAAGTATGGTCGTCCTCTGCTGGGATGTACTATTAAACCTAAATTGGGGTTATCCGCTAAAAACTATG

>T5549

GAGTACAAATTGACTTATTATACTCCTGAATATGAAACCAAAGATACTGATATCTTGGCAGCATTCCGAGTAACTCCTCAACCGGGAGTTCCACCTGAAGAAGCAGGGGCCGCGGTAGCTGCCGAATCCTCCACCGGTACATGGACAACCGTGTGGACCGATGGACTTACCAGCCTTGATCGTTACAAAGGGCGATGCTACAACATCGAGGCCGTTCCTGGCGAAGCAGATCAATACATCTGTTATGTAGCTTACCCTTTAGACCTTTTTGAAGAAGGTTCTGTTACCAACATGTTTACTTCCATTGTAGGAAATGTTTTTGGATTCAAAGCACTGCGTGCTCTACGTCTGGAAGATCTGCGAATCCCTGTTGCTTATGTTAAAACTTTCCAGGGCCCGCCTCATGGGATCCAAAGTGAGAGAGATAAATTGAACAAGTATGGTCGTCCTCTGCTGGGATGTACTATTAAACCTAAATTGGGGTTATCCGCTAAA

>T5550

GAGTACAAATTGACTTATTATACTCCTGAATATGAAACCAAAGATACTGATATCTTGGCAGCATTCCGAGTAACTCCTCAACCGGGAGTTCCACCTGAAGAAGCAGGGGCCGCGGTAGCTGCCGAATCCTCCACCGGTACATGGACAACCGTGTGGACCGATGGACTTACCAGCCTTGATCGTTACAAAGGGCGATGCTACAACATCGAGCCCGTTCCTGGCGAAGCAGATCAATACATCTGTTATGTAGCTTACCCTTTAGACCTTTTTGAAGAAGGTTCTGTTACCAACATGTTTACTTCCATTGTAGGAAATGTTTTTGGATTCAAAGCACTGCGTGCTCTACGTCTGGAAGATCTGCGAATCCCTGTTGCTTATGTTAAAACTTTCCAGGGTCCGCCTCATGGGATCCAAAGTGAGAGAGATAAATTGAACAAGTATGGTCGTCCTCTGCTGGGATGTACTATTAAACCTAAATTGGGGTTATCCGCTAAAAACTATG
