# Supplementary material for: Quality assessment of medicinal material Daqingye and Banlangen from Isatis tinctoria Fort. reveals widespread substitution with Strobilanthes species
Source: PLoS One. 2025 May 7;20(5):e0323084. doi: 10.1371/journal.pone.0323084 (PMC12058189; doi:10.1371/journal.pone.0323084)
Supplement: S5 File — (DOCX) [file pone.0323084.s005.docx]

**S5 File. Calibration curve of epigoitrin by HPLC method**

S6 Fig. Calibration curve of epigoitrin by HPLC method
