# Supplementary material for: Quality assessment of medicinal material Daqingye and Banlangen from Isatis tinctoria Fort. reveals widespread substitution with Strobilanthes species
Source: PLoS One. 2025 May 7;20(5):e0323084. doi: 10.1371/journal.pone.0323084 (PMC12058189; doi:10.1371/journal.pone.0323084)
Supplement: S6 File — (DOCX) [file pone.0323084.s006.docx]

**S6 File. Calibration curve of indirubin by HPLC method**

S7 Fig. Calibration curve of indirubin by HPLC method
